# Notes on Michael Schülke’s pselaphine collections from China. – Tyrini. I. genera *Labomimus* Sharp, *Linan* Hlaváč and *Pselaphodes* Westwood (Coleoptera, Staphylinidae, Pselaphinae)

**DOI:** 10.3897/zookeys.251.4099

**Published:** 2012-12-18

**Authors:** Zi-Wei Yin, Li-Zhen Li

**Affiliations:** 1 Department of Biology, College of Life and Environmental Sciences, Shanghai Normal University, Shanghai, 200234, P. R. China

**Keywords:** Staphylinidae, Pselaphinae, taxonomy, new species, new combination, China

## Abstract

This paper is the first of a series that deals with Dr. Michael Schülke’s collection of Pselaphinae from China. The tyrine genera *Labomimus* Sharp, *Linan* Hlaváč and *Pselaphodes* Westwood are chosen for the first part. The study revealed fourteen new species, all described and illustrated: *Labomimus cognatus* Yin & Li, **sp. n.** (Yunnan), *Labomimus dabashanus* Yin & Li, **sp. n.** (Hubei, Shaanxi), *Labomimus mirus* Yin & Li, **sp. n.** (Yunnan), *Labomimus paratorus* Yin & Li, **sp. n.** (Shaanxi), *Labomimus sarculus* Yin & Li, **sp. n.** (Yunnan), *Labomimus schuelkei* Yin & Li, **sp. n.** (Shaanxi), *Labomimus vespertilio* Yin & Li, **sp. n.** (Yunnan), *Linan tendothorax* Yin & Li, **sp. n.** (Yunnan), *Pselaphodes distincticornis* Yin & Li, **sp. n.** (Yunnan), *Pselaphodes erlangshanus* Yin & Li, **sp. n.** (Sichuan), *Pselaphodes flexus* Yin & Li, **sp. n.** (Yunnan), *Pselaphodes tibialis* Yin & Li, **sp. n.** (Yunnan), *Pselaphodes venustus* Yin & Li, **sp. n.** (Yunnan) and *Pselaphodes zhongdianus* Yin & Li, **sp. n.** (Yunnan). *Pselaphodes jizushanus* Yin, Li & Zhao is recorded from a new locality in Yunnan and its aedeagus is newly illustrated; new province records for *Pselaphodes nomurai* Yin, Li & Zhao is provided. *Labomimus torus* (Yin, Li & Zhao), **comb. n.** is moved from *Pselaphodes* after an examination of the holotype. Species represented only by unassociated females are listed with label data.

## Introduction

The Asian species of the *Pselaphodes* complex of genera (sensu Hlaváč 2002) belonging to the tribe Tyrini are most diverse in the east to southeast Oriental region (Hlaváč and Chandler 2005). Despite recent works (Hlaváč 2002, Hlaváč and Nomura 2001, Bekchiev 2010, Yin et al. 2010, 2011a, 2011b, 2011c, 2012a, 2012b, Yin and Li in press) describing a number of new genera and new species from that region, the complex is considered still inadequately studied at both the generic and species levels.

Recent access to the pselaphine collection of M. Schülke, which contains specimens collected during several expeditions by M. Schülke (Berlin, staphylinidologist) and D. W. Wrase (Berlin, carabidologist) to China, provided us an opportunity to work on a large number of yet undescribed tyrine species from that country. In this paper, members of the genera *Labomimus* Sharp, *Linan* Hlaváč and *Pselaphodes* Westwood are treated. The results are fourteen new species, one new record of known species and one new combination; sixteen species are represented only by female specimens, and are listed with their collecting data for future study. This information is reported herein.

## Material and methods

The material referred to in this study is housed in the following public institution and private collection:

**SNUC** Insect Collection of Shanghai Normal University, Shanghai (Z.-W. Yin)

**cSch** private collection M. Schülke, Berlin

The Michael Schülke collection will eventually be moved to Museum für Naturkunde, Berlin (**MNB**).

Labels of the examined material are quoted verbatim if not mentioned otherwise; a slash (/) is used to separate lines on the same label, and a double slash (//) is used to separate different labels.

Holotype bears the following label: ‘HOLOTYPE [red] / *xxx xxx* [genus name, species name] / sp. n., Yin & Li / det., 2012, xxx [depository]’, and paratype bears a similar label except: ‘PARATYPE [yellow]’.

The terminology of the foveal system follows Chandler 2001, except for using ‘ventrite’ instead of ‘sternite’ when discussing the meso- and metathoracic structures.

Measurements are in millimeters. The following acronyms are used in the text: **AL**–length of the abdomen along the midline; **AW**–maximum width of the abdomen; **BL**–length of the body (= HL + PL + EL + AL); **EL**–length of the elytra along the sutural line; **EW**–maximum width of the elytra; **HL**–length of the head from the anterior clypeal margin to the occipital constriction; **HW**–width of the head across eyes; **PL**–length of the pronotum along the midline; **PW**–maximum width of the pronotum.

## Taxonomy

### Labomimus cognatus

Yin & Li sp. n.

urn:lsid:zoobank.org:act:77BAE919-26BC-4D78-8094-5EED63B6C5BC

http://species-id.net/wiki/Labomimus_cognatus

Figs 1A 2

#### Type material

(3 ♂♂, 3 ♀♀)**.** Holotype: ♂, labeled ‘CHINA Yunnan [CH07-11], Baoshan / Pref., Gaoligong Shan, m. Xiaoheishan / N.R., 35 km SE Tengchong, 2110 m, / 24°"50'16"N, 98°"45'43"E, decid forest, / litter, sifted, 30.V.2007, M. Schülke’ (cSch). Paratypes, 1 ♂, same label data as holotype (SNUC); 1 ♂, labeled ‘CHINA: Yunnan, Baoshan Pref., Gao- / ligong Shan, W pass, 32 km SE / Tengchong, 1600 m, 25°51'11"N, 98°44'27"E, cleft with devast. primary / forest, litter & mushr. sifted, 28.VIII. / 2009, leg. M. Schülke [CH09-14]’ (cSch); 1 ♀, same label data except ‘D.W. Wrase [14]’ (cSch); 1 ♀, same label data, except ‘W pass, 35 km / SE Tengchong, 2100 m (devast. prim. / dec. forest, litter, sifted) / 24°50'18"N, 98°45'43"E / 25-28.VIII.2009 D.W. Wrase [06]’ (SNUC); 1 ♀, same label data, except ‘litter, wood, mushrooms sifted, 25.VIII. / 2009, leg. M. Schülke [CH09-06]’ (cSch).

#### Diagnosis.

Reddish brown; length 3.18–3.48; postgenae nearly rounded; antennomeres IX–XI enlarged, IX–X modified in the male; pronotum with lateral margins moderately angularly expanded laterally; with short blunt metaventral processes; metacoxae spinose; aedeagus with symmetric median lobe.

#### Description.

**Figure 1.**
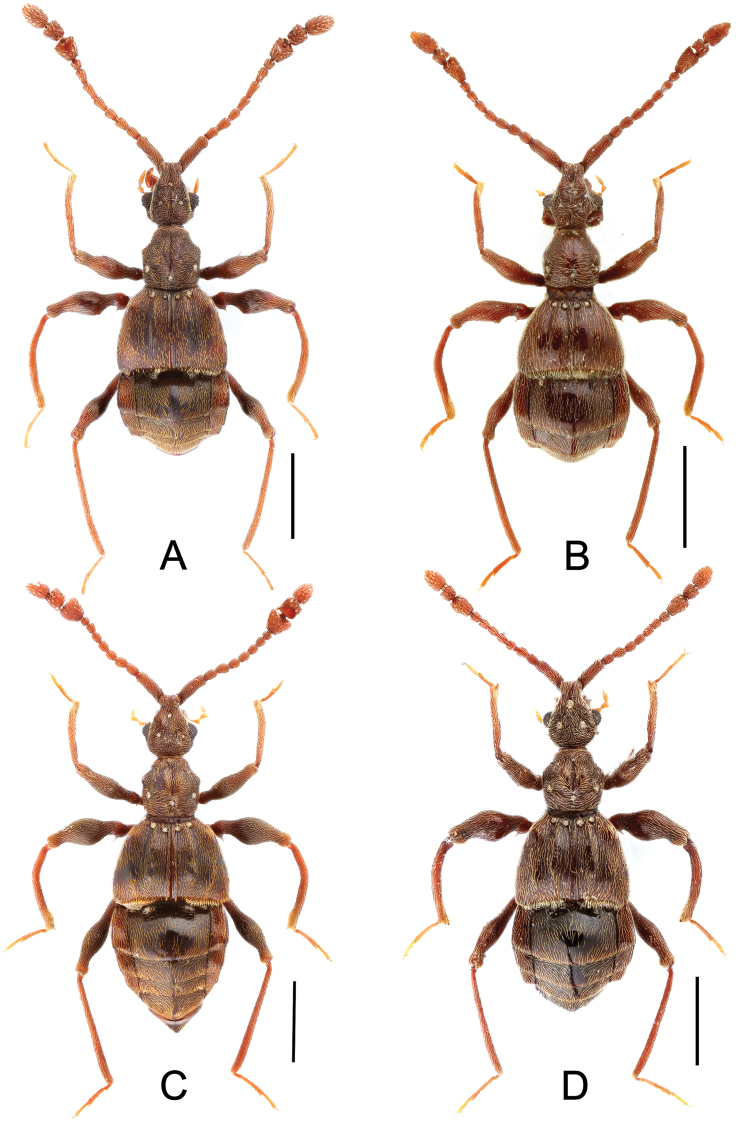
Male habitus of *Labomimus* spp. **A** *Labomimus cognatus* **B** *Labomimus dabashanus* **C** *Labomimus mirus* **D** *Labomimus paratorus*. Scales: 1.0 mm.

**Figure 2.**
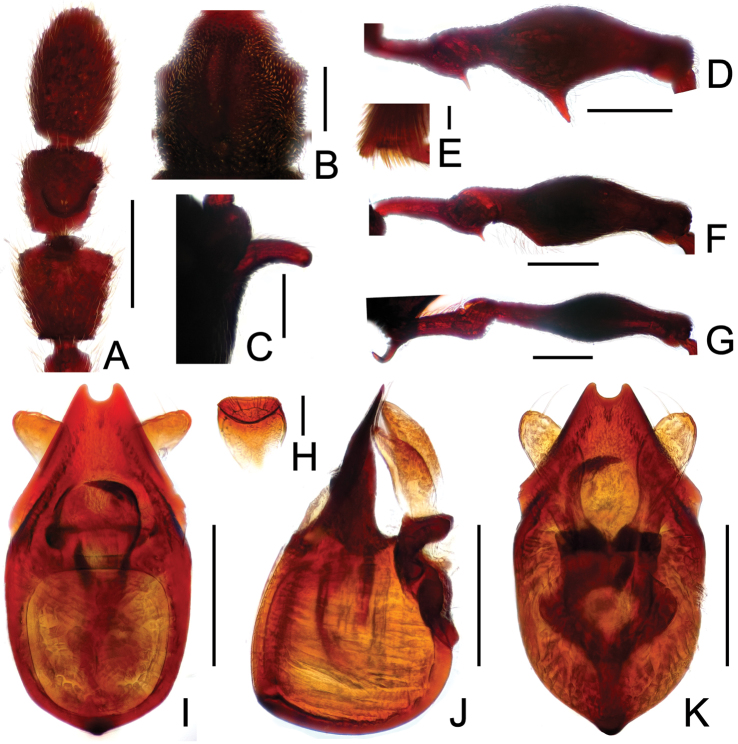
Diagnostic features of *Labomimus cognatus*. **A** antenna **B** pronotum **C** median meteventral process, in lateral view **D** protrochanter and profemur **E** apical portion of protibia **F** mesotrochanter and mesofemur **G** metacoxa, metatrochanter and metafemur **H** sternite IX **I** aedeagus, in dorsal view **J** same, in lateral view **K** same, in ventral view. Scales (mm): **A, B, D, F, G** = 0.3; **C, I, J, K** = 0.2; **H** = 0.1; **E** = 0.05.

Male (Fig. 1A). Length 3.18–3.41. Head longer than wide, HL 0.71–0.78, HW 0.65–0.71; eyes each composed of about 40 facets. Antennal clubs as in Fig. 2A. Pronotum (Fig. 2B) slightly longer than wide, PL 0.71–0.75, PW 0.66–0.71, with lateral margins moderately angularly expanded laterally. Elytra wider than long, EL 0.84–0.90, EW 1.12–1.35. Short metaventral processes with rounded apices (Fig. 2C). Protrochanters with small ventral spine, profemora with large ventral spine (Fig. 2D), protibiae with small apical tubercle (Fig. 2E); mesotrochanters (Fig. 2F) with tiny spine at ventral margin; metacoxae (Fig. 2G) with long hook-like protuberance at ventral margin, metatrochanters and metafemora simple. Abdomen broad at base and narrowed apically, AL 0.92–0.98, AW 1.23–1.37. Sternite IX as in Fig. 2H. Aedeagus length 0.50, with symmetric median lobe (Figs 2I–K).

Female. Similar to male in general; BL 3.25–3.48, HL 0.73–0.76, HW 0.65–0.66, PL 0.76–0.77, PW 0.71–0.72, EL 0.80–0.86, EW 1.34–1.41, AL 0.96–1.09, AW 1.41–1.46. Eyes each composed of about 27 facets. Antennae lacking modification; metaventral processes absent.

#### Comparative notes.

This is placed as a sister species of *Labomimus vespertilio* sp. n. described below. The two species share a similar general appearance, short metaventral process, similar placement of spines on the legs and a close aedeagal form. The two species can be readily separated by the smaller size, the symmetric antennomeres IX with a disc-like process and the median lobe has a narrower apex in *Labomimus cognatus*, while *Labomimus vespertilio* is larger in size, has strongly asymmetric antennomeres IX and has the aedeagus with median lobe much broader at apex.

#### Distribution.

Southwest China: Yunnan.

#### Biology.

Individuals were sifted from leaf litter in deciduous forests.

#### Etymology.

The Latin word ‘*cognatus*’ means ‘related’, indicating a close relationship between the new species and *Labomimus vespertilio* described below.

### Labomimus dabashanus

Yin & Li sp. n.

urn:lsid:zoobank.org:act:DBDF0116-0E4E-4C38-98E3-3609E077BE82

http://species-id.net/wiki/Labomimus_dabashanus

Figs 1B 3

#### Type material

(4 ♂♂, 5 ♀♀)**.** Holotype: ♂, labeled ‘CHINA: W-Hubei (Daba Shan) / pass E of Mt. Da Shengnongjia, / 12 km NW Muyuping, 31°30'N, / 110°21'E, 22.VII.2001, / leg. M. Schülke [C01-13E] // dry creek vaelly, mixed deciduous forest, dead wood, mushrooms, / moss, 1950-2050 m (sifted) / [C01-13E]’ (cSch). Paratypes: 2 ♂♂, 3 ♀♀, same label data as holotype (cSch, SNUC); 1 ♂, labeled ‘CHINA: S-Shaanxi (Qinling Shan) / pass on rd. Zhouzhi-Foping, / 105 km SW Xi’an, N-slope / 1880 m, 33°44'N, / 107°25'E / leg. M. Schülke [C01-03] // 4.VII.2001, / shady rockwall base, moist / (sifted) [C01-03]’ (cSch); 2 ♀♀, labeled ‘CHINA: W-Hubei (Daba Shan) / muntain range NE Muyuping, pass 12 km N Muyuping, / 31°32'N, 110°26'E, 2380 m, / leg. M. Schülke [C01-15] // 17.VII 2001, / N pass, N-slope with young deciduous forest, bank of / small creek, moss (sifted) [C01-15]’ (cSch). All types also bear the following labels: ‘Sammlung / M. Schülke / Berlin // M. SCHÜLKE Coll. / Staphylinidae, Pselaphinae / *Labomimus* sp. 13 / S. Nomura det., 2005’.

#### Diagnosis.

Reddish brown; length 2.36–2.96; postgenae broadly expanded laterally; antennomeres IX–XI enlarged, IX–X modified in the male; pronotum with round lateral margins; with long metaventral processes; metacoxae simple; aedeagus with asymmetric median lobe.

#### Description.

**Figure 3.**
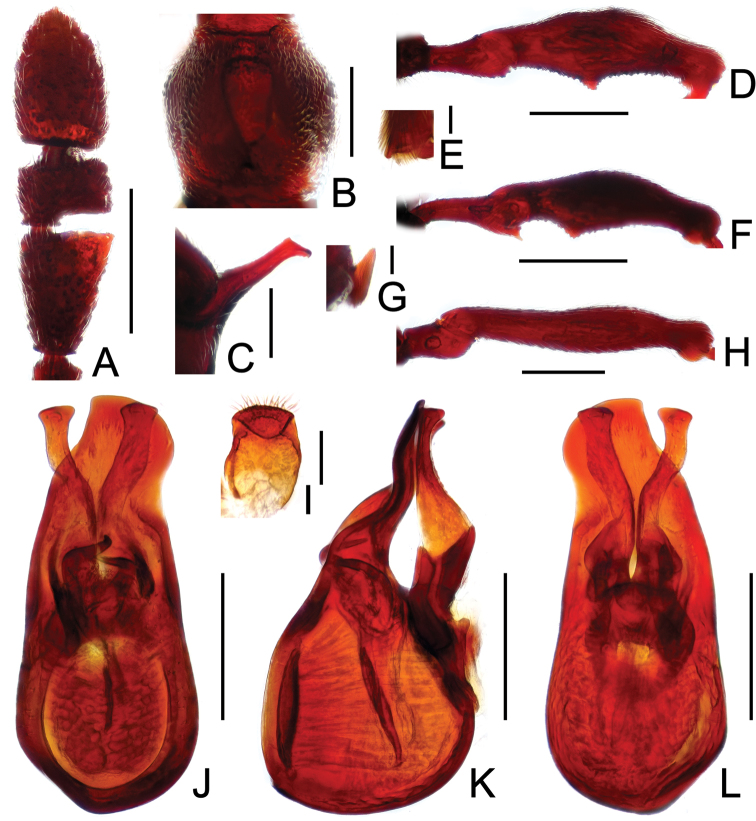
Diagnostic features of *Labomimus dabashanus*. **A** antenna **B** pronotum **C** median meteventral process, in lateral view **D** protrochanter and profemur **E** apical portion of protibia **F** mesotrochanter and mesofemur **G** metatrochanter and metafemur **H** metacoxa **I** sternite IX **J** aedeagus, in dorsal view **K** same, in lateral view **L** same, in ventral view. Scales (mm): **A, B, D, F, G** = 0.3; **C, J, K, L** = 0.2; **I** = 0.1; **E, H** = 0.05.

Male (Fig. 1B). Length 2.85–2.96. Head slightly longer than wide, HL 0.63–0.66, HW 0.60–0.62; eyes each composed of about 25 facets. Antennal clubs as in Fig. 3A. Pronotum (Fig. 3B) as long as wide, PL and PW 0.57–0.59, with round lateral margins. Elytra wider than long, EL 0.69–0.71, EW 1.09–1.10. Metaventral processes long, broadened apically with truncate apices (Fig. 3C). Profemora with short triangular ventral spine (Fig. 3D), protibiae with small apical spur (Fig. 3E); mesotrochanters (Fig. 3F) with small spine and mesofemora angularly protuberant ventrally; metacoxae (Fig. 3G) with short ventral protuberance, metatrochanters and metafemora (Fig. 3H) simple. Abdomen broad at base and narrowed apically, AL 0.96–1.00, AW 1.16–1.20. Sternite IX as in Fig. 3I. Aedeagus length 0.57, with asymmetric median lobe elongate (Figs 3J–L).

Female. Similar to male in general; BL 2.36–2.50, HL 0.56–0.58, HW 0.49–0.50, PL 0.51–0.52, PW 0.50–0.52, EL 0.57–0.60, EW 1.09–1.10, AL 0.72–0.80, AW 1.19–1.22. Eyes each composed of about 20 facets. Antennae lacking modification; metaventral processes absent.

#### Comparative notes.

The new species is closest to *Labomimus shibatai* K. Sawada, 1961 by sharing the laterally expanded postgenae and rounded pronotal lateral margins. Apart from the clearly different aedeagal form, the two species can be separated by the much smaller body size, the transverse antennomeres X, and the elongate antennomeres IX being angularly expanded at anteromedial margin in *Labomimus dabashanus*. *Labomimus shibatai* is much larger in size (3.5–3.8 mm), has elongate antennomeres X and enlarged and unmodified antennomeres IX.

#### Distribution.

Central China: Hubei; Norwest China: Shaanxi.

#### Biology.

Individuals were sifted from leaf litter and moss in deciduous forests.

#### Etymology.

Named after the type locality ‘Dabashan Mountain’.

### Labomimus mirus

Yin & Li sp. n.

urn:lsid:zoobank.org:act:B118D9BC-BFCD-4DFF-B508-39BB3AEEFFC7

http://species-id.net/wiki/Labomimus_mirus

Figs 1C 4

#### Type material

(6 ♂♂, 1 ♀)**.** Holotype: ♂, labeled ‘CHINA (N-Yunnan) Dali Bai Nat. / Aut. Pref., Diancang Shan, 3 km / W Dali old town, pine forest at / “cloud road”, night upper chair- / list station, 24°41.1"N, 100°06.8'E / 2650-2750 m (needle/leaf litter) / 1.IX.2003 Wrase [19C] // Sammlung / M. Schülke / Berlin // M. SCHÜLKE Coll. / Staphylinidae, Pselaphinae / *Labomimus* sp. 6 / S. Nomura det., 2005’ (cSch). Paratypes: 1 ♂, same label data, except with additional label as ‘[C03-19A] pine needles, moss / (dry) in ditches, mushrooms, / 30.VIII.2003, leg. M. Schülke’ (SNUC); 4 ♂♂, labeled ‘CHINA: Yunnan [CH07-09], / Dali Bai Auton. Pref., Diancang Shan 45 / km NW Dali, 2730 m, 26°01'20"N, 99°53'17"E, creek valley, pines, ferns, / sifted, 29.V.2007, M. Schülke’ (cSch, SNUC); 1 ♀, same label data, except ‘25°41'09"N, 100°06'32"E, 3000- / 3200 m, cleft in mixed forest, litter, / debirs sifted, 27.V.2007, M. Schülke’(cSch).

#### Diagnosis.

Reddish brown; length 3.36–3.75; postgenae rounded; antennomeres IX–XI enlarged, IX–X modified in the male; pronotum with lateral margins moderately angulate laterally; with short sharp metaventral processes; metacoxae simple; aedeagus with median lobe nearly symmetric.

#### Description.

Male (Fig. 1C). Length 3.41–3.75. Head slightly longer than wide, HL 0.71–0.72, HW 0.65–0.68; eyes each composed of about 45 facets. Antennal clubs as in Fig. 4A. Pronotum (Fig. 4B) about as long as wide, PL 0.70–0.73, PW 0.65–0.71, with roundly angulate lateral margins. Elytra wider than long, EL 0.87–0.93, EW 1.38–1.40. Metaventral processes short, narrowed from base toward apex (Fig. 4C). Protrochanters with small ventral spine, profemora with large thick ventral spine (Fig. 4D); mesotrochanters (Fig. 4E) with small spine at ventral margin, mesotibiae (Fig. 4F) with short blunt apical tubercle; metatrochanters and metafemora (Fig. 4G) simple. Abdomen broad at base and narrowed apically, AL 1.13–1.37, AW 1.35–1.43. Sternite IX as in Fig. 4H. Aedeagus length 0.67, with broad median lobe elongate nearly symmetric (Figs 3I–K).

Female. Similar to male in general; BL 3.36, HL 0.71, HW 0.64, PL 0.66, PW 0.64, EL 0.74, EW 1.21, AL 1.25, 1.43. Eyes each composed of about 35 facets. Antennae lacking modification; metaventral processes absent.

#### Comparative notes.

The postgenae of the head being rounded and not laterally expanded quickly separates this species from *Labomimus sichuanicus* Hlaváč, *Labomimus schuelkei* sp. n. described below, *Labomimus dabashanus* and *Labomimus shibatai*. From the rest of the members of the genus, *Labomimus mirus* can be readily recognized by its characteristic antennal modification and aedeagus.

#### Distribution.

Southwest China: Yunnan.

#### Biology.

Species were sifted from various kinds of leaf litter and moss in mixed forests.

#### Etymology.

Tha Latin word ‘*mirus*’ means ‘extraodinary, remarkable’, referring to the unique antennal modification and aedeagal form of this species.

**Figure:**
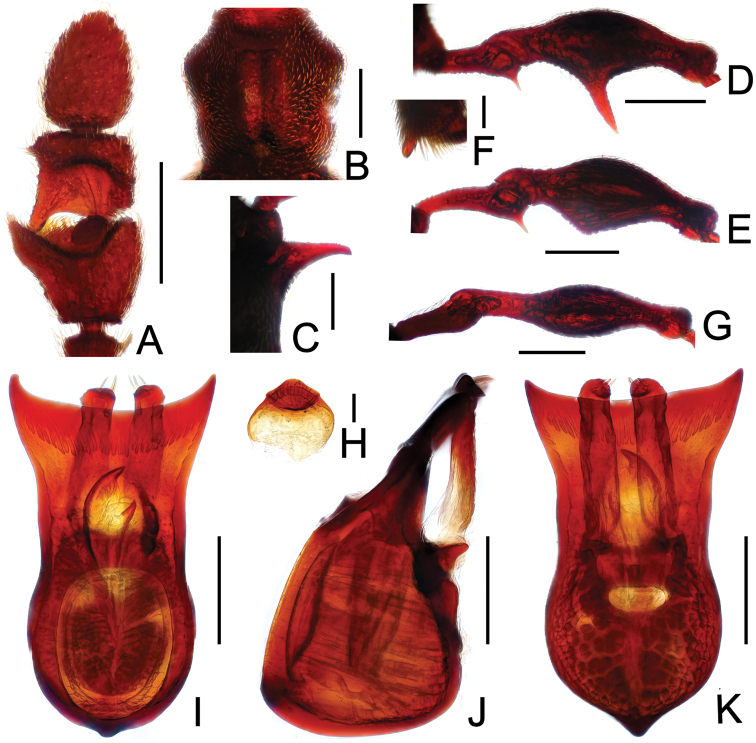
**Figure 4.** Diagnostic features of *Labomimus mirus*. **A** antenna **B** pronotum **C** median meteventral process, in lateral view **D** protrochanter and profemur **E** mesotrochanter and mesofemur **F** apical portion of mesotibia **G** metatrochanter and metafemur **H** sternite IX **I** aedeagus, in dorsal view **J** same, in lateral view **K** same, in ventral view. Scales (mm): **A, B, D, E, G** = 0.3; **C, I, J, K** = 0.2; **H** = 0.1; **F** = 0.05.

### Labomimus paratorus

Yin & Li sp. n.

urn:lsid:zoobank.org:act:AE6BCF63-0112-4CAE-9D52-FBFE72490A1B

http://species-id.net/wiki/Labomimus_paratorus

Figs 1D 5

#### Type material

(3 ♂♂)**.** Holotype: ♂, labeled ‘China: Shaanxi, Qin Ling Shan / 108.47E, 33.51N, Mountain W / pass at Autoroute km 70, 47 km / 2500-2600 m, sifted / 26.-27.08.1995, leg. M. Schülke // Sammlung / M. Schülke / Berlin // M. SCHÜLKE Coll. / Staphylinidae, Pselaphinae / *Labomimus* sp. 5 / S. Nomura det., 2005’ (cSch). Paratypes: 1 ♂, same label data as holotype, with additional identification label ‘*Labomimus* Sharp sp. / det. Brachat 2. 99’ (SNUC); 1 ♂, labeled ‘CHINA (S-Shaanxi) Qingling Shan / mount. range W pass on rd. Xi’an / - Shagoujie, 45 km SSE Xi’an, / 33°52'N, 108°46'E, 2675 m / (N-slope, *Abies*, *Betula*, *Larix*, / subalp. meadows, along road) / 25.VII.2001 Wrase [20] // Sammlung / M. Schülke / Berlin // M. SCHÜLKE Coll. / Staphylinidae, Pselaphinae / *Labomimus* sp. 14 / S. Nomura det., 2005’ (cSch).

#### Diagnosis.

Reddish brown; length 3.55–3.87; postgenae rounded; antennomeres IX–XI enlarged, IX modified in the male; pronotum with lateral margins slightly angularly expanded laterally; metaventral processes roundly broadened apically; metacoxae simple; aedeagus with asymmetric median lobe.

#### Description.

**Figure 5.**
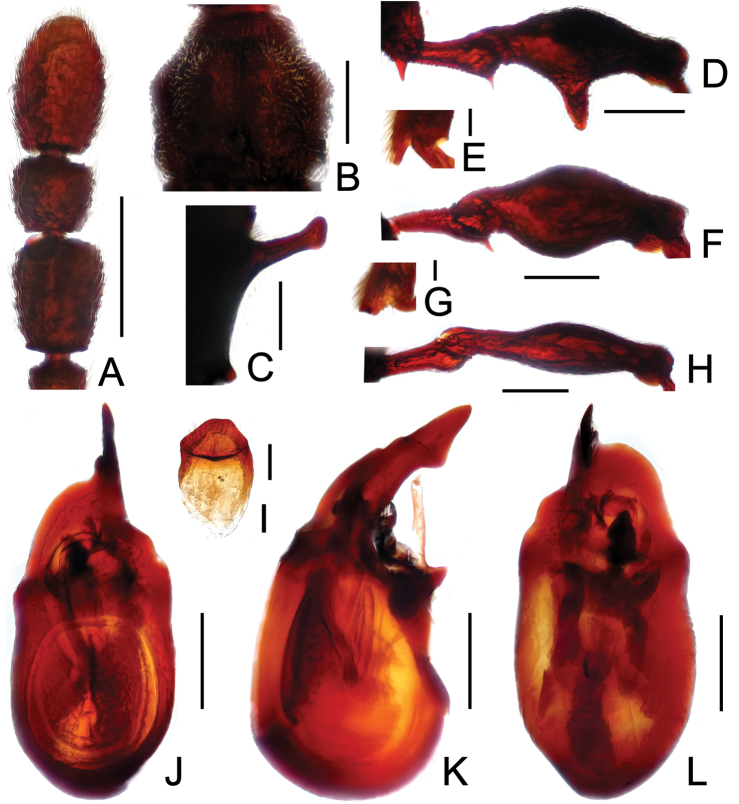
Diagnostic features of *Labomimus paratorus*. **A** antenna **B** pronotum **C** median meteventral process, in lateral view **D** procoxa, protrochanter and profemur **E** apical portion of protibia **F** mesotrochanter and mesofemur **G** apical portion of mesotibia **H** metatrochanter and metafemur **I** sternite IX **J** aedeagus, in dorsal view **K** same, in lateral view **L** same, in ventral view. Scales (mm): **A, B, D, F, H** = 0.3; **C, J, K, L** = 0.2; **I** = 0.1; **E, G** = 0.05.

Male (Fig. 1D). Length 3.55–3.87. Head longer than wide, HL 0.74–0.76, HW 0.66–0.69; eyes each composed of about 40 facets. Antennal clubs as in Fig. 5A. Pronotum (Fig. 5B) about as long as wide, PL 0.68–0.70, PW 0.69–0.71, with lateral margins slightly angularly expanded. Elytra wider than long, EL 0.92–0.93, EW 1.25–1.28. Metaventral processes moderately elongate, apically roundly enlarged (Fig. 5C). Procoxae with short thick ventral spine, protrochanters with small ventral spine, profemora with large blunt spine at ventral margin (Fig. 5D), protibiae (Fig. 5E) with small apical tubercle; mesotrochanters (Fig. 5F) with small spine at ventral margin, mesotibiae (Fig. 5G) with short truncate apical tubercle; metatrochanters and metafemora (Fig. 5H) simple. Abdomen broad at base and narrowed apically, AL 1.21–1.48, AW 1.40–1.43. Sternite IX as in Fig. 5I. Aedeagus length 0.81, median lobe elongate, asymmetric (Figs 5J–L).

**Female.** Unknown.

#### Comparative notes.

A reexamination of the holotype of *Pselaphodes torus* Yin, Li & Zhao clearly showed the presence of a median metaventral fovea in that species, a character state that is shared by *Labomimus*, *Linan*,and *Indophodes* Hlaváč of the *Pselaphodes*-complex. Combined with the short tarsomeres II not extending to beneath the III, the distinct frontal and vertexal foveae, and the presence of a pronotal median antebasal fovea, *Pselaphodes torus* is here moved to *Labomimus*, comb. n. *Labomimus paratorus* is placed closest to *Labomimus torus*, they share a similar general appearance, the placement of spines on the legs, and even similar aedeagal form. The two species can be separated only by reddish-brown body coloration and the short apical projections of pro- and mesotibiae in *Labomimus paratorus*, in contrast *Labomimus torus* has black body coloration and much longer apical projections of the first two pairs of tibiae.

#### Distribution.

Norwest China: Shaanxi.

#### Biology.

Individuals were sifted from leaf litter of mixed forests.

#### Etymology.

The species name indicates a close relationship to *Labomimus torus*.

### Labomimus sarculus

Yin & Li sp. n.

urn:lsid:zoobank.org:act:88F4D2D6-C9B6-4B74-9CC4-472673FC4EBE

http://species-id.net/wiki/Labomimus_sarculus

Figs 6A 7

#### Type material

(2 ♂♂)**.** Holotype: ♂, labeled ‘CHINA: Yunnan, Baoshan Pref., Gao- / ligong Shan, 32 km SE Tengchong,/ 2150-2250 m, 24°51-53'N, 98°45'E, / devast. prim. and second. forest litter, / dead wood, mushrooms sifted, 26.VIII. / 2009, leg. M. Schülke [CH09-08/09]’ (cSch). Paratype: 1 ♂, same label data as holotype (cSch).

#### Diagnosis.

Reddish brown; length 2.89–3.02; postgenae nearly rounded; antennomeres IX–XI enlarged, IX–X modified in the male; pronotum with lateral margins slightly angularly expanded laterally; metaventral processes short and sharp; metacoxae with large hook-like ventral protuberance; aedeagus with symmetric median lobe.

#### Description.

**Figure 6.**
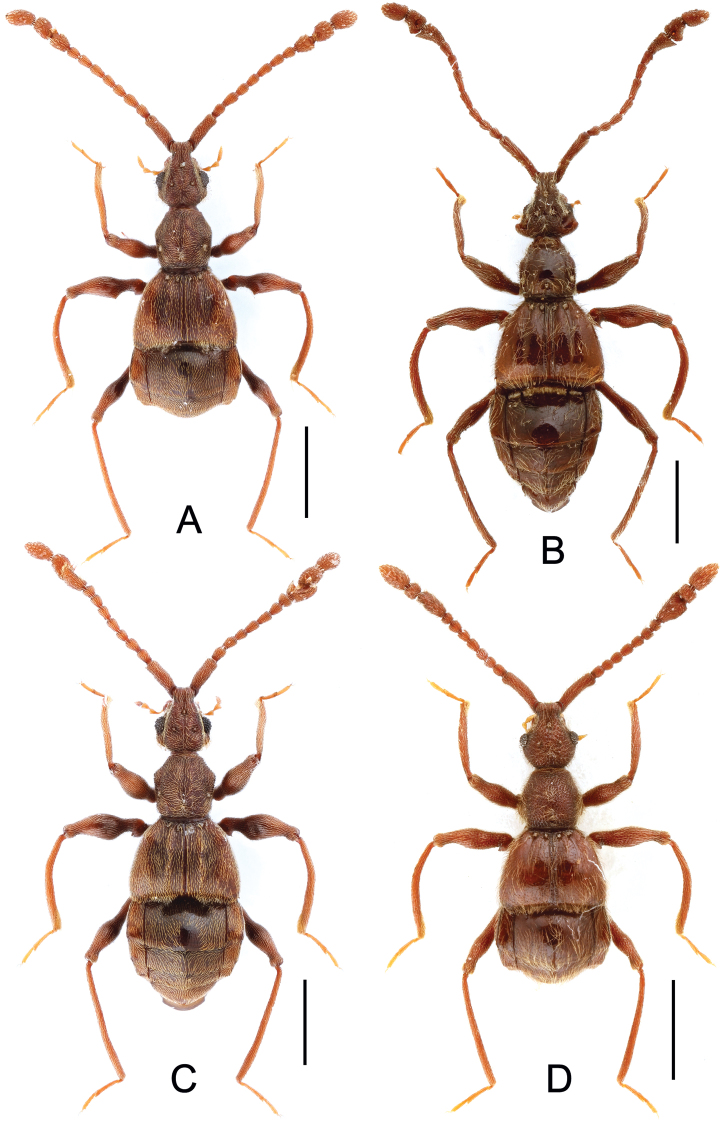
Male habitus of *Labomimus* and *Linan* spp. **A** *Labomimus sarculus* **B** *Labomimus schuelkei* **C** *Labomimus vespertilio* **D** *Linan tendothorax*. Scales: 1.0 mm.

**Figure 7.**
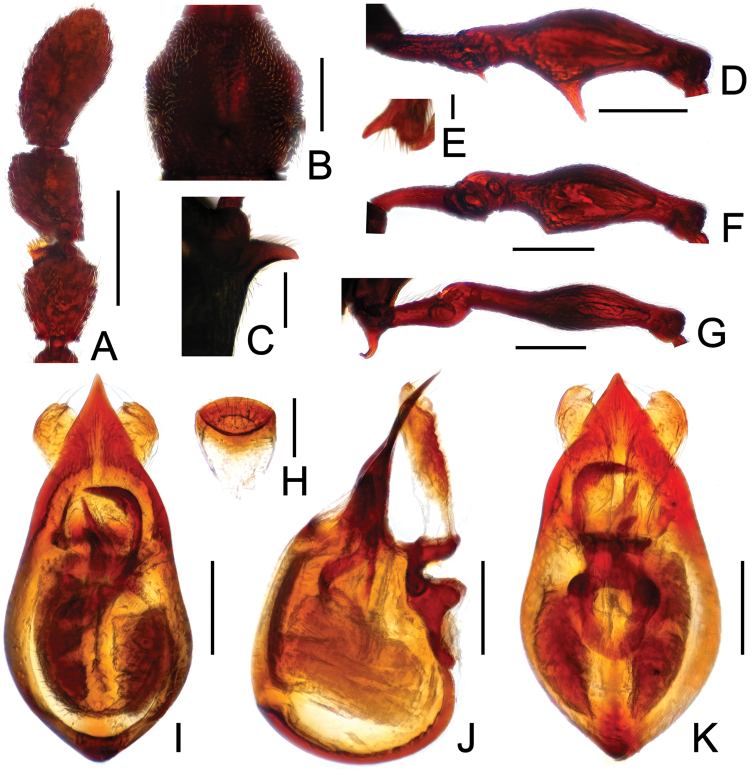
Diagnostic features of *Labomimus sarculus*. **A** antenna **B** pronotum **C** median meteventral process, in lateral view **D** protrochanter and profemur **E** apical portion of protibia **F** mesotrochanter and mesofemur **G** metacoxa, metatrochanter and metafemur **H** sternite IX **I** aedeagus, in dorsal view **J** same, in lateral view **K** same, in ventral view. Scales (mm): **A, B, D, F, G** = 0.3; **C, I, J, K** = 0.2; **H** = 0.1; **E** = 0.05.

Male (Fig. 6A). Length 2.89–3.02. Head longer than wide, HL 0.70–0.71, HW 0.66–0.69; eyes each composed of about 25 facets. Antennal clubs as in Fig. 7A. Pronotum (Fig. 7B) slightly longer than wide, PL 0.68–0.70, PW 0.64–0.65, with lateral margins slightly angularly expanded. Elytra wider than long, EL 0.75–0.76, EW 1.18–1.19. Metaventral processes short, narrowed apically with pointed apices (Fig. 7C). Protrochanters with small ventral spine, profemora with large sharp spine at ventral margin (Fig. 7D), protibiae (Fig. 7E) with distinct preapical apur; mesotrochanters (Fig. 7F) with indistinct tiny spine at ventral margin; metacoxae (Fig. 7G) with big hook-like protuberance at ventral margin, metatrochanters and metafemora simple. Abdomen broad at base and narrowed apically, AL 0.76–0.85, AW 1.23–1.26. Sternite IX as in Fig. 7H. Aedeagus length 0.60, median lobe symmetric, narrowed from base toward apex (Figs 7I–K).

**Female.** Unknown.

#### Comparative notes.

This species is placed near *Labomimus cognatus* and *Labomimus vespertilio* described below by sharing a similar habitus, the short median metaventral process and the protuberant metacoxae. *Labomimus sarculus* can be readily separated from both species by the unique modified antennomeres IX, longer apical spur of protibiae and the aedeagus has the median lobe medially straight at the apex, not concave.

#### Distribution.

Southwest China: Yunnan.

#### Biology.

Individuals were sifted from leaf litter and mixed deadwood and mushrooms in a devastated primary and secondary forest.

#### Etymology.

The Latin word ‘*sarculus*’ means ‘a hoe’, referring to the uniquely modified antennomeres IX.

### Labomimus schuelkei

Yin & Li sp. n.

urn:lsid:zoobank.org:act:1616C90A-14ED-4941-A128-5AFA6E28028E

http://species-id.net/wiki/Labomimus_schuelkei

Figs 6B 8

#### Type material

(1 ♂)**.** Holotype: ♂, labeled ‘China: Shaanxi, Qin Ling Shan / 110.06E, 34.27N / Hua Shan Mt. N Valley, 1200- / 1400 m, 118 km E Xi’an, sifted / 18.-20.08.1995, leg. M. Schülke // *Eulasinus* Sharp sp. / det. Brachat 2. 99 // Sammlung / M. Schülke / Berlin // M. SCHÜLKE Coll. / Staphylinidae, Pselaphinae / *Labomimus* sp. 4 / S. Nomura det., 2005’ (cSch).

#### Diagnosis.

Reddish brown; length 3.92; postgenae strongly expanded laterally; antennomeres IX–XI enlarged, IX–X modified in the male; pronotum with lateral margins nearly rounded; metaventral processes short; metacoxae simple; aedeagus with asymmetric median lobe.

#### Description.

**Figure 8.**
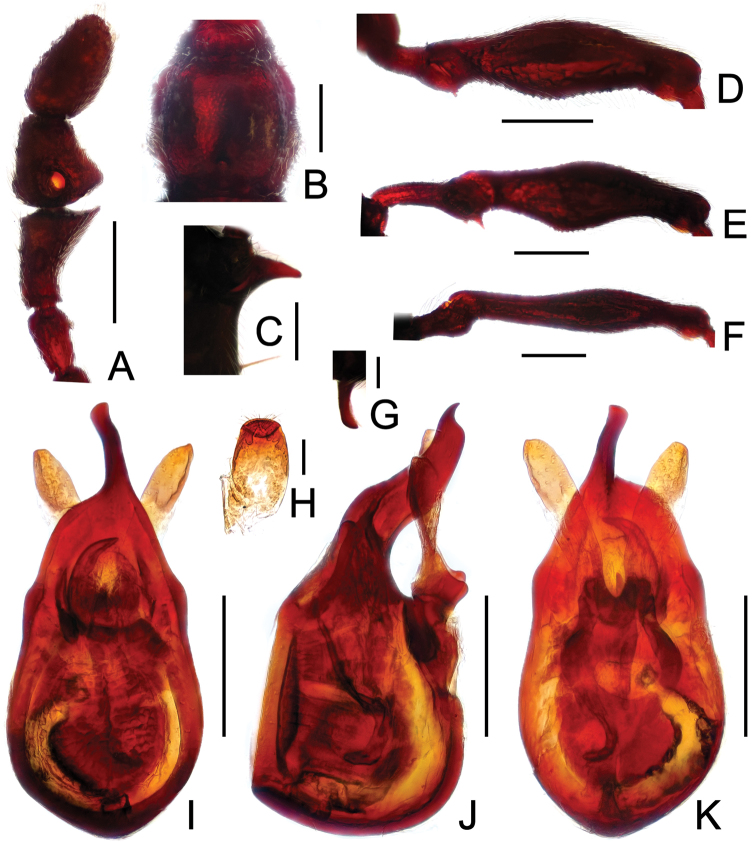
Diagnostic features of *Labomimus schuelkei*. **A** antenna **B** pronotum **C** median meteventral process, in lateral view **D** protrochanter and profemur **E** mesotrochanter and mesofemur **F** metatrochanter and metafemur G metacoxa **H** sternite IX **I** aedeagus, in dorsal view **J** same, in lateral view **K** same, in ventral view. Scales (mm): **A, B, D, E, F** = 0.3; **C, I, J,** K = 0.2; **H, G** = 0.1.

Male (Fig. 6B). Length 3.92. Head longer than wide, HL 0.89, HW 0.78; eyes each composed of about 20 facets. Antennal clubs as in Fig. 8A. Pronotum (Fig. 8B) slightly longer than wide, PL 0.76, PW 0.70, with lateral margins nearly rounded. Elytra wider than long, EL 0.89, EW 1.34. Metaventral processes short, apically narrowed (Fig. 8C). Protrochanters with tiny ventral spine, profemora simple (Fig. 8D); mesotrochanters (Fig. 8E) with one big spine and one smaller spine at ventral margin; metacoxae with elongate protuberance (Fig. 8F), metatrochanters and metafemora (Fig. 8G) simple. Abdomen broad at base and narrowed apically, AL 1.38, AW 1.37. Sternite IX as in Fig. 8H. Aedeagus length 0.62, asymmetric median lobe narrow (Figs 8I–K).

**Female.** Unknown.

#### Comparative notes.

*Labomimus schuelkei* is placed close to *Labomimus sichuanicus* by sharing the postgenae being largely expanded laterally with a thickened posterior margin, and the strongly elongate antennomeres V–VIII. The two species can be readily separated by the large body size, the strongly modified antennomeres IX–X, and the aedeagus with the median lobe narrow dorsal-ventrally in *Labomimus schuelkei*, while *Labomimus sichuanicus* is much smaller (3.05–3.20 mm), has simple antennomeres IX–X, and has the aedeagus with a much broader median lobe (Hlaváč et al. 2000).

#### Distribution.

Northwest China: Shaanxi.

#### Biology.

Probably sifted from leaf litter in a forest.

#### Etymology.

Named after Michael Schülke, a well-known specialist in Staphylinidae, who kindly provided all the material used in this paper.

### Labomimus vespertilio

Yin & Li sp. n.

urn:lsid:zoobank.org:act:16AA692E-CA85-4705-A6A7-FB71A99A0C06

http://species-id.net/wiki/Labomimus_vespertilio

Figs 6C 9

#### Type material

(2 ♂♂, 5 ♀♀)**.** Holotype: ♂, labeled ‘CHINA: Yunnan, Dali Bai Aut. Pref., / mount. range E Weishan, 12 km NE / Weishan, 25°17'02-15"N, 100°22' / 22-30"E, 2630-2660 m, scrub with / pines and bamboo, litter sifted, 15.IX. / 2009. leg. M. Schülke [CH09-54]’ (cSch). Paratypes: 1 ♂, 5 ♀♀, same label data as holotype (cSch, SNUC).

#### Diagnosis.

Reddish brown; length 3.34–3.52; postgenae nearly rounded; antennomeres IX–XI enlarged, VIII–X modified in the male; pronotum with lateral margins moderately angularly expanded laterally; with short blunt metaventral processes; metacoxae spinose; aedeagus with symmetric median lobe.

#### Description.

**Figure 9.**
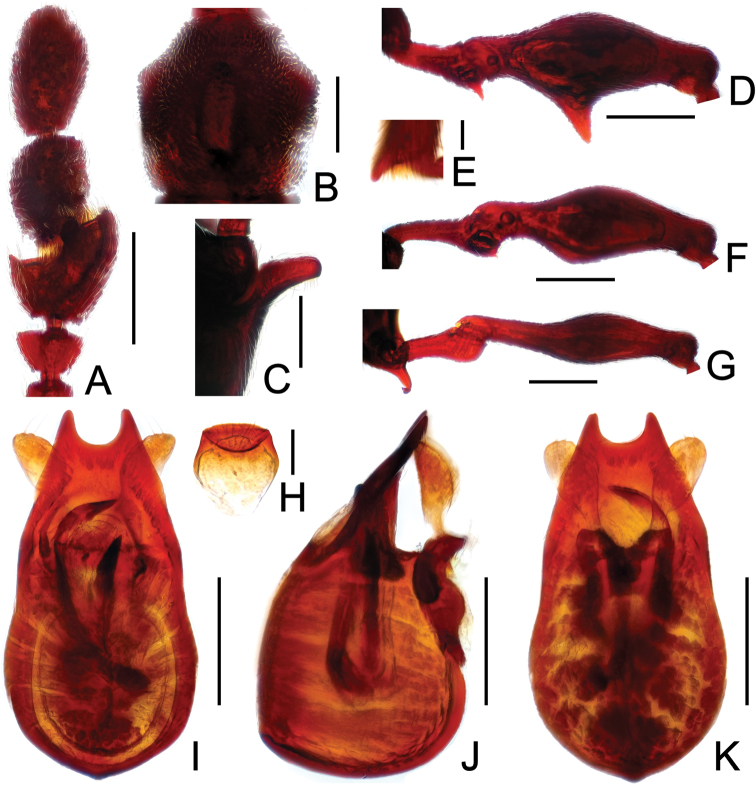
Diagnostic features of *Labomimus vespertilio*. **A** antenna **B** pronotum **C** median meteventral process, in lateral view **D** protrochanter and profemur **E** apical portion of protibia **F** mesotrochanter and mesofemur **G** metacoxa, metatrochanter and metafemur **H** sternite IX **I** aedeagus, in dorsal view **J** same, in lateral view **K** same, in ventral view. Scales (mm): **A, B, D, F, G** = 0.3; **C, I, J, K** = 0.2; **H** = 0.1; **E** = 0.05.

Male (Fig. 6C). Length 3.34–3.52. Head longer than wide, HL 0.70–0.75, HW 0.63–0.65; eyes each composed of about 30 facets. Antennal clubs as in Fig. 9A. Pronotum (Fig. 9B) slightly longer than wide, PL 0.71–0.74, PW 0.65–0.70, with lateral margins moderately angularly expanded laterally. Elytra wider than long, EL 0.75–0.81, EW 1.23–1.28. Short metaventral processes with rounded apices (Fig. 9C). Protrochanters with small ventral spine, profemora with large ventral spine (Fig. 9D), protibiae with distinct apical tubercle (Fig. 9E); mesotrochanters (Fig. 9F) with tiny spine at ventral margin; metacoxae (Fig. 9G) with long hook-like protuberance at ventral margin, metatrochanters and metafemora simple. Abdomen broad at base and narrowed apically, AL 1.18–1.22, AW 1.28–1.35. Sternite IX as in Fig. 9H. Aedeagus length 0.56, with symmetric median lobe (Figs 9I–K).

Female. Similar to male in general; BL 3.34–3.40, HL 0.72–0.73, HW 0.61–0.62, PL 0.72–0.73, PW 0.68–0.70, EL 0.73–0.74, EW 1.28–1.29, AL 1.17–1.20, AW 1.38–1.41. Eyes each composed of about 25 facets. Antennae lacking modification; metaventral processes absent.

#### Comparative notes.

This is placed as a sister species of *Labomimus cognatus*, sharing with it a number of character states (see comparative notes under *Labomimus cognatus*). The two species can be separated by the larger body size, the strongly asymmetric antennomeres IX, and the aedeagus with much broader apex in *Labomimus vespertilio*, while *Labomimus cognatus* is smaller in body size, has symmetric antennomeres IX with a disc-like process, and has the aedeagus with a much narrower apex. Other than the aforementioned characters, the two species also share with *Labomimus sarculus* the lateral rows of dense setae extending from frontal rostrum base to head base, and the three species seem toform a small species-complex. For separation of *Labomimus sarculus* from *Labomimus cognatus* and *Labomimus vespertilio* see the comparative notes under that species.

#### Distribution.

Southwest China: Yunnan.

#### Biology.

Adults were from sifted leaf litter in a scrub forest with pines and bamboo.

#### Etymology.

The Latin word ‘*vespertilio*’ means ‘a bat’, referring to the bat-like apical part of the aedeagal median lobe.

### Linan tendothorax

Yin & Li sp. n.

urn:lsid:zoobank.org:act:3B08AD2E-62ED-4031-96EE-EB853063EA33

http://species-id.net/wiki/Linan_tendothorax

Figs 6D 10

#### Type material

(3 ♂♂, 14 ♀♀)**.** Holotype: ♂, labeled ‘CHINA: Yunnan, Lincang Pref., Xue / Shan, 11 km ENE Lincang, 2510 m, / 23°55'01"N, 100°11'17.5"E, second. / pine forest with Rhodod., small cleft with water, litter & mushrooms sifted, / 10.IX.2009, leg. M. Schülke [CH09-39]’ (cSch). Paratypes: 1 ♂, 7 ♀♀, same label data as holotype (cSch, SNUC); 1 ♀, same label data, except ‘D.W. Wrase’ (cSch); 3 ♀♀, labeled ‘CHINA: Yunnan, Lincang Pref., / Laobei Shan, Wei Bo Shan pass, / 24°08'16"N, 99°42'53"E, 2375 m, / creek valley, devastated second. / decid. forest, litter & moss sifted, 8.IX.2009, leg. M. Schülke [CH09-35]’ (cSch); 3 ♀♀, labeled ‘CHINA (Yunnan) Lincang Pref., / Wuliang Shan, old pass road, W-side, / 2200 m (small creek valley with primary / forest remnant, litter, debris sifted) / 24°42'58.6"N, 100°29'52"E / 12.IX.2009 D.W.Wrase’ (cSch); 1 ♂, labeled ‘CHINA: Yunnan, Pu’er Pref., / Ailao Shan, 37 km NW Jingdong, / 24°45'12"N, 100°41'24.5"E, 2300 m, / devastated forest remnant, litter & / dead wood sifted, 13.IX.2009, / leg. M. Schülke [CH09-48]’ (cSch).

#### Diagnosis.

Reddish brown; length 2.80–2.95; postgenae rounded; antennomeres IX–XI enlarged, IX modified in the male; pronotum with lateral margins roundly expanded basolaterally; with short blunt metaventral processes; metacoxae spinose; aedeagus with asymmetric median lobe.

#### Description.

**Figure 10.**
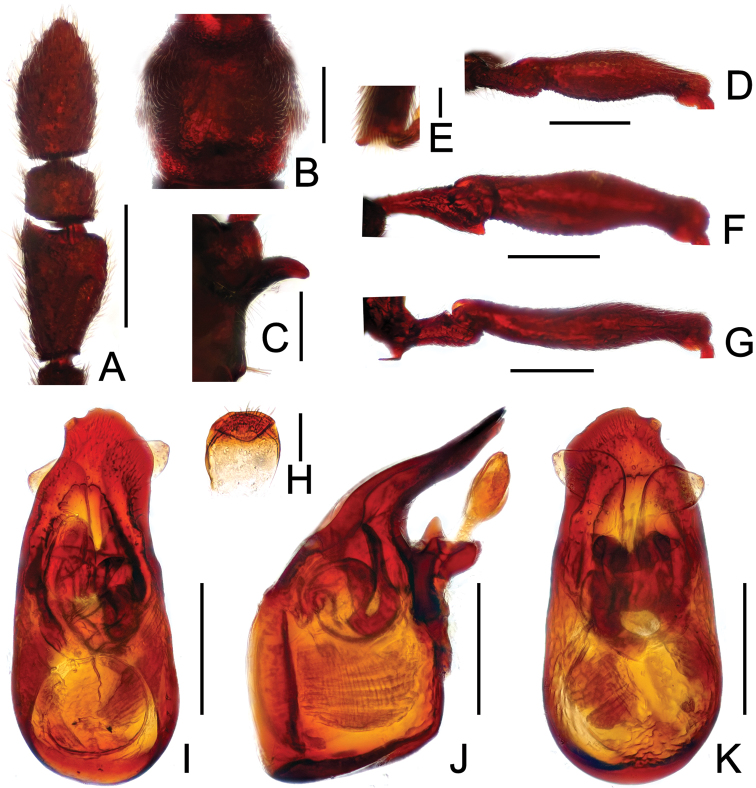
Diagnostic features of *Linan tendothorax*. **A** antenna **B** pronotum **C** median meteventral process, in lateral view **D** protrochanter and profemur **E** apical portion of protibia **F** mesotrochanter and mesofemur **G** metacoxa, metatrochanter and metafemur **H** sternite IX **I** aedeagus, in dorsal view **J** same, in lateral view **K** same, in ventral view. Scales (mm): **A, B, D, F, G** = 0.3; **C, I, J, K** = 0.2; **H** = 0.1; **E** = 0.05.

Male (Fig. 6D). Length 2.80–2.95. Head longer than wide, HL 0.65–0.66, HW 0.59–0.60; eyes each composed of about 17 facets. Antennal clubs as in Fig. 10A. Pronotum (Fig. 10B) about as long as wide, PL 0.61–0.62, PW 0.59–0.60, with round lateral margins. Elytra wider than long, EL 0.71–0.74, EW 1.08–1.09. Short metaventral processes with rounded apices (Fig. 10C). Protrochanters and profemora simple (Fig. 10D), protibiae with distinct small apical tubercle (Fig. 10E); mesotrochanters (Fig. 10F) with blunt triangular spine at ventral margin; metacoxae (Fig. 10G) with hook-like protuberance at ventral margin, metatrochanters and metafemora simple. Abdomen broad at base and narrowed apically, AL 0.83–0.93, AW 1.14–1.19. Sternite IX as in Fig. 10H. Aedeagus length 0.60, with asymmetric median lobe (Figs 10I–K).

Female. Similar to male in general; BL 2.81–2.92, HL 0.64–0.66, HW 0.58–0.59, PL 0.60–0.62, PW 0.60–0.62, EL 0.70–0.72, EW 1.16–1.19, AL 0.87–0.92, AW 1.22–1.25. Eyes each composed of about 16 facets. Antennae lacking modification; metaventral processes absent.

#### Comparative notes.

*Linan tendothorax* is placed as a member of the *Labomimus cardialis* species-group (sensu Yin et al. 2011b) based on its strongly modified antennomeres IX–X. It can be separated from all known *Linan* species by the unique pronotum that is roundly expanded laterally at the basolateral margins.

#### Distribution.

Southwest China: Yunnan.

#### Biology.

Individuals were sifted from mixed litter, moss, debris and dead wood in primary or secondary deciduous forests.

#### Etymology.

The species name is combined from the Latin stem ‘*tend*’ and Greek word ‘*thorax*’, referring to the unique basolaterally extended pronotum of the new species.

### Pselaphodes distincticornis

Yin & Li sp. n.

urn:lsid:zoobank.org:act:68CE4309-47C1-40AD-A7B1-2EA84A398A4F

http://species-id.net/wiki/Pselaphodes_distincticornis

Figs 11A 12

#### Type material

(2 ♂♂, 1 ♀)**.** Holotype: ♂, labeled ‘CHINA: Yunnan, Dali Bai Aut. Pref. / 36 km N Dali, ruderal pasture wtih / pines and shrubs, 26°01'20"N, / 100°08'14"E, 2158 m, litter sifted / under pines and shrubs, 24.VIII.2009, / leg. M. Schülke [CH09-04]’ (cSch). Paratype: 1 ♂, same label data as holotype (SNUC); 1 ♀, same label data sa holotype, except ‘D.W. Wrase [04]’.

#### Diagnosis.

Reddish brown; length 2.74–2.88; postgenae rounded; antennomeres IX–XI enlarged, IX modified in the male; pronotum with lateral margins roundly expanded laterally; with long thick metaventral processes; metacoxae simple; aedeagus with asymmetric median lobe elongate.

#### Description.

**Figure 11.**
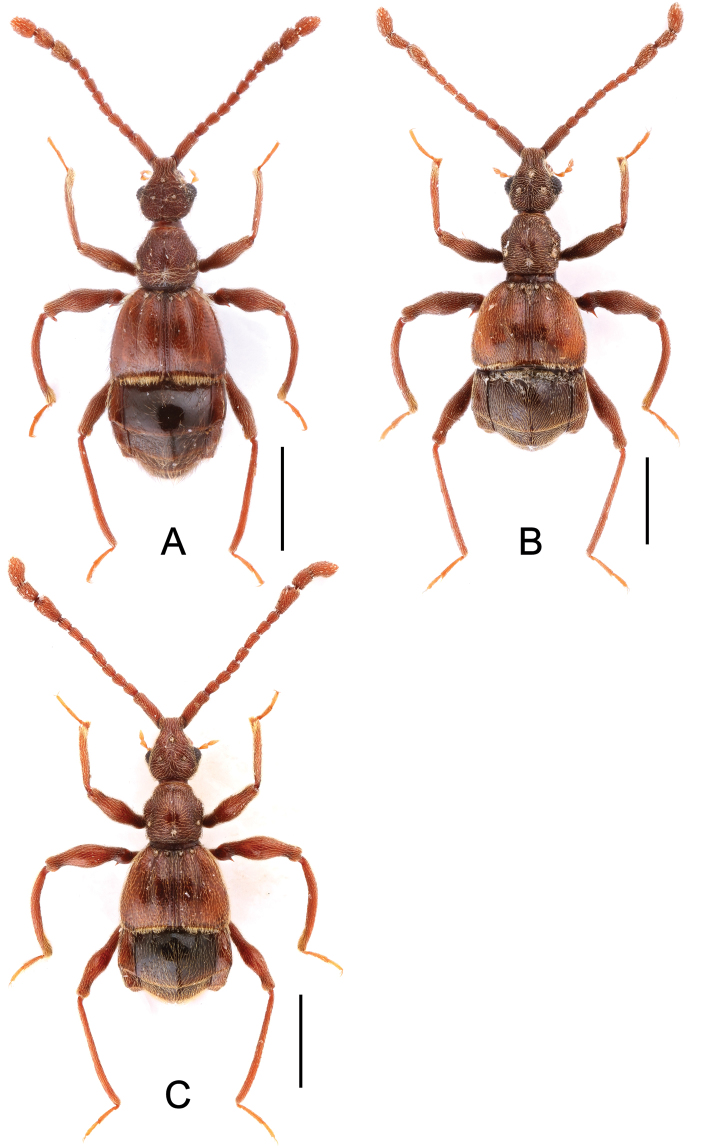
Male habitus of *Pselaphodes* spp. **A** *Pselaphodes distincticornis* **B** *Pselaphodes erlangshanus* **C** *Pselaphodes flexus*. Scales: 1.0 mm.

**Figure 12.**
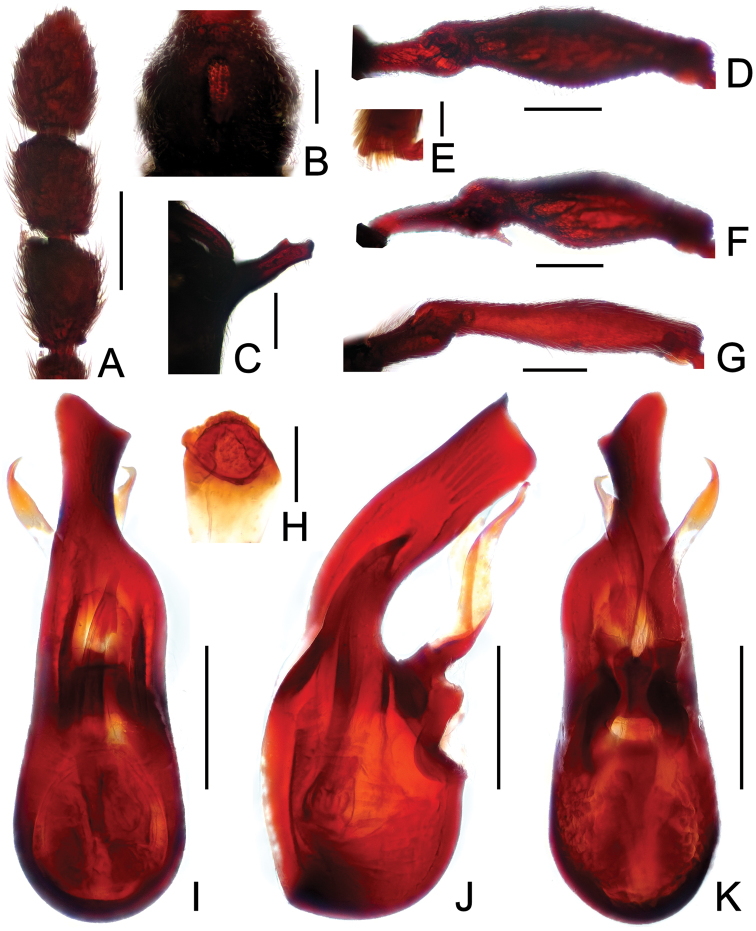
Diagnostic features of *Pselaphodes distincticornis*. **A** antenna **B** pronotum **C** median meteventral process, in lateral view **D** protrochanter and profemur **E** apical portion of protibia **F** mesotrochanter and mesofemur **G** metatrochanter and metafemur **H** sternite IX **I** aedeagus, in dorsal view **J** same, in lateral view **K** same, in ventral view. Scales (mm): **A, B, C, D, F, G, I, J, K** = 0.2; **H** = 0.1; **E** = 0.05.

Male (Fig. 11A). Length 2.74–2.88. Head longer than wide, HL 0.59–0.60, HW 0.58–0.59; eyes each composed of about 40 facets. Antennal clubs as in Fig. 12A. Pronotum (Fig. 12B) about as long as wide, PL 0.58–0.59, PW 0.58–0.60, with round lateral margins. Elytra wider than long, EL 0.72–0.73, EW 1.11–1.12. Long metaventral processes with truncate apices (Fig. 12C). Protrochanters and profemora simple (Fig. 12D), protibiae with short apical tubercle (Fig. 12E); mesotrochanters (Fig. 12F) with small spine at ventral margin; metatrochanters and metafemora (Fig. 12G) simple. Abdomen broad at base and narrowed apically, AL 0.85–0.96, AW 1.11–1.14. Sternite IX as in Fig. 12H. Aedeagus length 0.71, with asymmetric median lobe distinctively elongate (Figs 12I–K).

**Female.** Similar to male in general; BL 2.79, HL 0.64, HW 0.58, PL 0.59, PW 0.59, EL 0.62, EW 1.16, AL 0.94, AW 1.22. Eyes each composed of about 30 facets. Metaventral processes absent.

#### Comparative notes.

The unmodified antennal clubs are shared in *Pselaphodes fengtingae* Yin, Li et Zhao (Zhejiang, Jiangxi) and *Pselaphodes parvus* Yin, Li et Zhao (Guizhou). *Pselaphodes distincticornis* can be separated from bothspeciesby the larger size, the simple protrochanters and profemora, and the distinctively asymmetric and elongate median lobe of the aedeagus. Both *Pselaphodes fengtingae* and *Pselaphodes parvus* have the protrochanters with a small ventral spine, and the profemora with a larger spine at the ventral margin, and have the aedeagus with an asymmetric but much shorter median lobe.

#### Distribution.

Southwest China: Yunnan.

#### Biology.

Species were sifted from leaf litter under pines and shrubs in a ruderal pasture.

#### Etymology.

Species name combined from Latin stems ‘*distinct*’ and ‘*corn*’, referring to the large median metaventral processes of the new species.

### Pselaphodes erlangshanus

Yin & Li sp. n.

urn:lsid:zoobank.org:act:ABAFBD58-6774-4127-A007-652A5C24A7A1

http://species-id.net/wiki/Pselaphodes_erlangshanus

Figs 11B 13

#### Type material

(2 ♂♂)**.** Holotype: ♂, labeled ‘CHINA: W-Sichuan 1999 / Ganzi Tibet. Aut. Pref., Luding Co. / W Erlanshan-pass, 2600 m / 7 km SSE Luding, 29°51'N, / 102°15'E, Laub+Nadelstreu, Pilze / 22. VI., leg. M. Schülke // Sammlung / M. Schülke / Berlin // *Labomimus* Sharp sp. / det. Brachat 2.09 // M. SCHÜLKE Coll. / Staphylinidae, Pselaphinae / *Lasinus* sp. 1 / S. Nomura det., 2005’ (cSch). Paratype: 1 ♂, same label data as holotype, except ‘det. Brachat 4.01’ (cSch).

#### Diagnosis.

Reddish brown; length 3.29–3.78; postgenae nearly rounded; antennomeres IX–XI enlarged, IX modified in the male; pronotum with lateral margins slightly angularly expanded laterally; with long metaventral processes apically narrowed; metacoxae simple; aedeagus with asymmetric median lobe.

#### Description.

**Figure 13.**
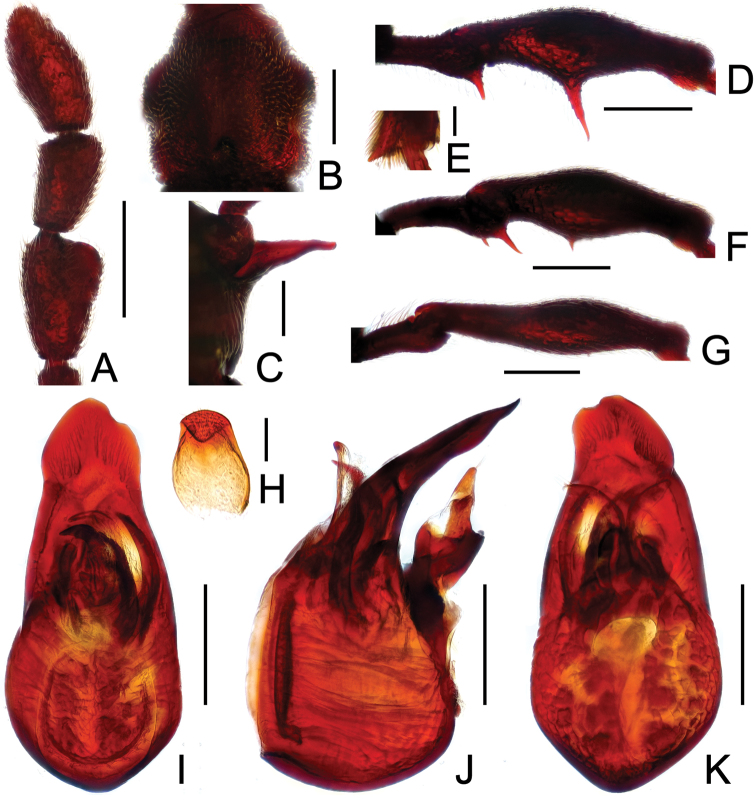
Diagnostic features of *Pselaphodes erlangshanus*. **A** antenna **B** pronotum **C** median meteventral process, in lateral view **D** protrochanter and profemur **E** apical portion of protibia **F** mesotrochanter and mesofemur **G** metatrochanter and metafemur **H** sternite IX **I** aedeagus, in dorsal view **J** same, in lateral view **K** same, in ventral view. Scales (mm): **A, B, D, F, G** = 0.3; **C, I, J, K** = 0.2; **H** = 0.1; **E** = 0.05.

Male (Fig. 11B). Length 3.29–3.78. Head longer than wide, HL 0.74–0.78, HW 0.65–0.68; eyes each composed of about 30 facets. Antennal clubs as in Fig. 13A. Pronotum (Fig. 13B) about as long as wide, PL 0.70–0.72, PW 0.68–0.69, with lateral margins slightly angularly expanded laterally. Elytra wider than long, EL 0.87–0.90, EW 1.34–1.37. Long metaventral (Fig. 13C) processes thick at base, narrowed apically. Protrochanters with small ventral spine, profemora with long sharp spine at ventral margin (Fig. 13D), protibiae with distinct short apical spur (Fig. 13E); mesotrochanters with large and another much smaller spine at ventral margin, mesofemora with tiny ventral spine (Fig. 13F); metatrochanters and metafemora (Fig. 13G) simple. Abdomen broad at base and narrowed apically, AL 0.98–1.00, AW 1.35–1.38. Sternite IX as in Fig. 13H. Aedeagus length 0.65, with asymmetric median lobe (Figs 13I–K).

**Female.** Unknown.

#### Comparative notes.

This species may be related to *Pselaphodes flexus* and *Pselaphodes zhongdianus* (both described below) by sharing a similar general habitus, elongate antennomeres IX–XI, and a somewhat similar aedeagal form. *Pselaphodes erlangshanus* can be readily separated from *Pselaphodes flexus* by the larger size, the mesotrochanters with two ventral spines, and quite different form of the metaventral processes. The form of the antennomeres IX and aedeagus provide a quick separation of the new species from *Pselaphodes zhongdianus*.

#### Distribution.

Southwest China: Sichuan.

#### Biology.

Individuals were sifted from leaf litter in a coniferous forest.

#### Etymology.

Named after the type locality.

### Pselaphodes flexus

Yin & Li sp. n.

urn:lsid:zoobank.org:act:00C65770-8F24-4C21-A177-070255101FD4

http://species-id.net/wiki/Pselaphodes_flexus

Figs 11C 14

#### Type material

(1 ♂, 7 ♀♀)**.** Holotype: ♂, labeled ‘CHINA (N-Yunnan) Lijiang Naxi / Aut. Co., E Yulongxue Shan, / 30 km N Lijiang, 2800-2900 m / 25°09.0'N, 100°14.9'E (creek / valley, secondary mixed forest) / 13.VIII.2003 Wrase [01] // Sammlung / M. Schülke / Berlin // M. SCHÜLKE Coll. / Staphylinidae, Pselaphinae / *Labomimus* sp. 9 / S. Nomura det., 2005’ (cSch). Paratypes: 3 ♀♀, same label data as holotype, except ‘*Labomimus* sp. 7 / S. Nomura det., 2005’; 4 ♀♀, same label data, except ‘13.VIII.2003, M. Schülke, (cSch, SNUC).

#### Diagnosis.

Reddish brown; length 2.87; postgenae rounded; antennomeres IX–XI enlarged, IX modified in the male; pronotum with lateral margins slightly angularly expanded laterally; with long metaventral processes apically narrowed; metacoxae simple; aedeagus with asymmetric median lobe.

#### Description.

**Figure 14.**
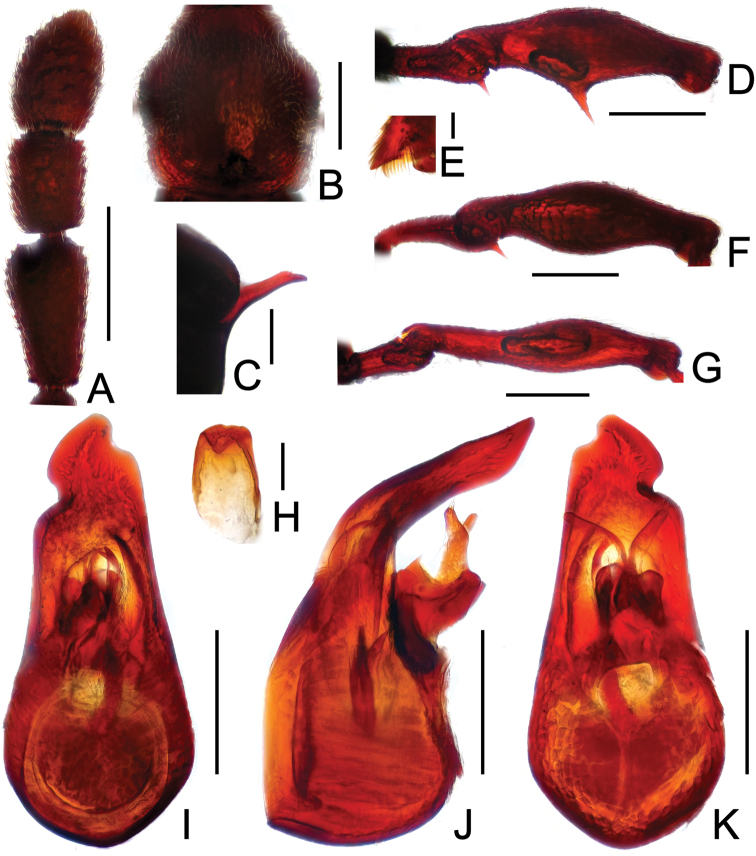
Diagnostic features of *Pselaphodes flexus*. **A** antenna **B** pronotum **C** median meteventral process, in lateral view **D** protrochanter and profemur **E** apical portion of protibia **F** mesotrochanter and mesofemur **G** metatrochanter and metafemur **H** sternite IX **I** aedeagus, in dorsal view **J** same, in lateral view **K** same, in ventral view. Scales (mm): **A, B, D, F, G** = 0.3; **C, I, J, K** = 0.2; **H** = 0.1; **E** = 0.05.

Male (Fig. 11C). Length 2.87. Head longer than wide, HL 0.66, HW 0.60; eyes each composed of about 25 facets. Antennal clubs as in Fig. 14A. Pronotum (Fig. 14B) about as long as wide, PL 0.65, PW 0.63, with lateral margins slightly angularly expanded laterally. Elytra wider than long, EL 0.81, EW 1.12. Long metaventral (Fig. 14C) processes narrowed apically. Protrochanters with small ventral spine, profemora with big sharp spine at ventral margin (Fig. 14D), protibiae with broad triangular spur (Fig. 14E); mesotrochanters with small spine at ventral margin, mesofemora simple (Fig. 14F); metatrochanters and metafemora (Fig. 14G) simple. Abdomen broad at base and narrowed apically, AL 0.75, AW 1.23. Sternite IX as in Fig. 14H. Aedeagus length 0.60, with asymmetric median lobe (Figs 14I–K).

**Female.** Unknown.

#### Comparative notes.

As discussed above, this species may be related to *Pselaphodes erlangshanus* and *Pselaphodes zhongdianus* by sharing a similar general habitus, elongate antennomeres IX–XI, and a somewhat similar aedeagal form. *Pselaphodes flexus* can be separated from *Pselaphodes erlangshanus* by the smaller size, the mesotrochanters with single ventral spine, and much thinner metaventral process. The nearly symmetrically cylindrical antennomeres IX of *Pselaphodes flexus* readily separate it from *Pselaphodes zhongdianus*, whose antennomeres IX are strongly roundly and projecting anterolaterally.

#### Distribution.

Southwest China: Yunnan.

#### Biology.

The individual was sifted from a secondary mixed forest in a ravine.

#### Etymology.

The Latin word ‘*flexus*’ means ‘curved, bent, twisting’, referring to the curved terminal antennomere of the new species.

### Pselaphodes jizushanus

Yin, Li & Zhao

http://species-id.net/wiki/Pselaphodes_jizushanus

Figs 15A 16

Pselaphodes jizushanus Yin, Li & Zhao, 2011a: 471.

#### Additional material examined.

1 ♂, labeled ‘CHINA: Yunnan [CH07-15], Baoshan / Pref., Gaoligong Shan, 29 km ESE / Tengchong, 24°55'37"N, / 98°45'09"E, / 2350 m, dev. decid. forest, litter, wood, / fungi sifted, 1.VI.2007, M. Schülke’ (cSch).

#### Diagnosis and description.

Yin et al. 2011: 471; Figs 15A, 16. Measurements: BL 3.10, HL 0.63, HW 0.59, PL 0.63, PW 0.62, EL 0.80, EW 1.14, AL 1.04, AW 1.12; eyes each composed of about 40 facets. Aedeagus length 0.51, with slightly asymmetrical median lobe (Figs 16J–L).

#### Distribution.

Southwest China: Yunnan.

**Notes.** This species was originally described based on a single male (Type-locality: Jizushan Mountain, ca. 25°57'37"N, 100°22'44"E, alt. 2400 m). The aedeagus of the holotype was unfortunately lost. Here we record a second male specimen of this species from Tengchong, Gaoligong Mountain, about 200 km southwest from the type locality, and have illustrated its aedeagus.

**Figure 15.**
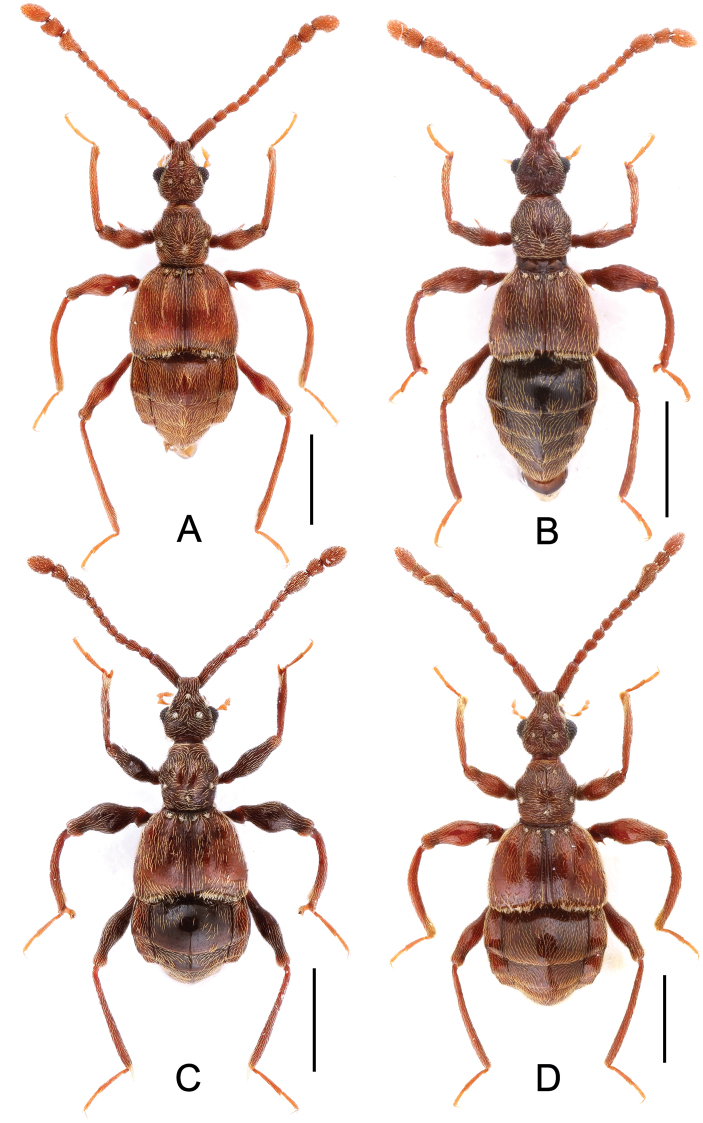
Male habitus of *Pselaphodes* spp. **A** *Pselaphodes jizushanus* **B** *Pselaphodes tibialis* **C** *Pselaphodes venustus* **D** *Pselaphodes zhongdianus*. Scales: 1.0 mm.

**Figure 16.**
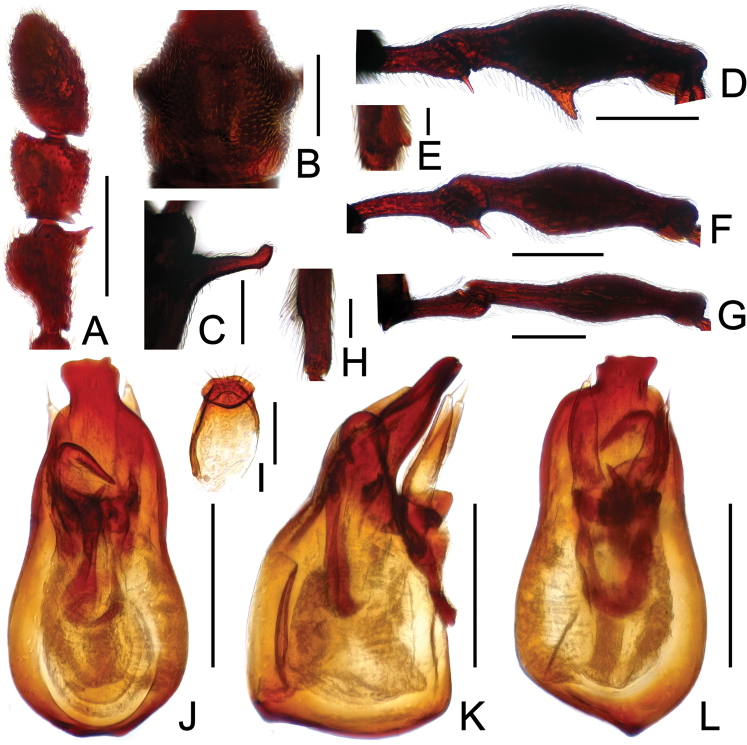
Diagnostic features of *Pselaphodes jizushanus*. **A** antenna **B** pronotum **C** median meteventral process, in lateral view **D** protrochanter and profemur **E** apical portion of protibia **F** mesotrochanter and mesofemur **G** metacoxa, metatrochanter and metafemur **H** apical portion of metatibia **I** sternite IX **J** aedeagus, in dorsal view **K** same, in lateral view **L** same, in ventral view. Scales (mm): **A, B, D, F, G, J, K, L** = 0.3; **C** = 0.2; **H, I** = 0.1; **E** = 0.05.

### Pselaphodes nomurai

Yin, Li & Zhao

http://species-id.net/wiki/Pselaphodes_nomurai

Pselaphodes nomurai Yin, Li & Zhao, 2010: 21.

#### Additional material examined

(20 ♂♂, 18 ♀♀)**.** 2 ♂♂, labeled ‘CHINA: Border Shaanxi - / Sichuan (Daba Shan), pass 20 km / SSE Zhenping, 1700-1800 m, / 31°44'N, 109°35'E, 9.VII.2001, / leg. M. Schülke [C01-07] // young dry mixed forest, / field edge, small creek valley / moss sifted [C01-07]’; 3 ♂♂, 2 ♀♀, same label data, except ’12.VII.2001’; 11 ♂♂, 13 ♀♀, same label data, except ‘Wrase [07]’; 3 ♂♂, 1 ♀, labeled ‘CHINA: S-Shaanxi (Qingling Shan) / pass on rd. Zhouzhi-Foping, / 105 km SW Xi’an, N-slpoe, / 1700 m, 33°46'N, 107°58'E / leg. M. Schülke [C01-02] // 3.VII.2001, / small creek valley, mixed / deciduous forest, moss / (sifted) [C01-02]’; 1 ♂, labeled ‘CHINA: S-Shaanxi (Qingling Shan) / river bank above Houzhenzi, / 115 km WSW Xi’an, / 1450 m, 33°50'N, 107°47'E, leg. M. Schülke [C01-06] // 5.VII.2001, / gravel bank (floating), / mixed deciduous forest, moss, / mushrooms (sifted) [C01-06]’ (all above specimens bear the following label: ‘M. SCHÜLKE Coll. / Staphylinidae, Pselaphinae / *Labomimus* sp. 11 / S. Nomura det., 2005’); 2 ♀♀, labeled ‘CHINA: W-Hubei (Daba Shan) / creek valley 8 km NW Muyuping, / 31°29'N, 110°22'E, 1550- / 1650 m, 18.VII.2001, / leg. M. Schülke [C01-16A] // creek valley, deciduous / forest, moss / (sifted) [C01-16A] // M. SCHÜLKE Coll. / Staphylinidae, Pselaphinae / *Labomimus* sp. 12 / S. Nomura det., 2005’ (all cSch). All specimens also bear the following label: ‘Sammlung / M. Schülke / Berlin // *Pselaphodes nomurai* / Yin, Li & Zhao, 2010 / det. Yin & Li, 2012’.

#### Diagnosis and description.

Yin et al., 2011: 479 (key); Yin et al., 2010: 21.

#### Distribution.

Southwest China: Shaanxi; Central China: Henan, Hubei (new provincial record).

### Pselaphodes tibialis

Yin & Li sp. n.

urn:lsid:zoobank.org:act:AAEA7BAB-9AB1-45E3-B63F-83E97F51FD79

http://species-id.net/wiki/Pselaphodes_tibialis

Figs 15B 17

#### Type material

(2 ♂♂)**.** Holotype: ♂, labeled ‘CHINA: Yunnan [CH07-09], / Dali Bai Aut. Pref., Diancang Shan 45 / km NW Dali, 2730 m, 26°01'20"N, 99°53'17"E, creek valley, pines, ferns, / sifted, 29.V.2007, M. Schülke’ (cSch). Paratype: 1 ♂, same label data as holotype (cSch).

#### Diagnosis.

Reddish brown; length 2.52–2.58; postgenae slightly angulate posterolaterally; antennomeres IX–XI enlarged, IX modified in the male; pronotum with lateral margins slightly angularly expanded laterally; with metaventral processes apically enlarged; metacoxae simple; aedeagus with asymmetric median lobe.

#### Description.

**Figure 17.**
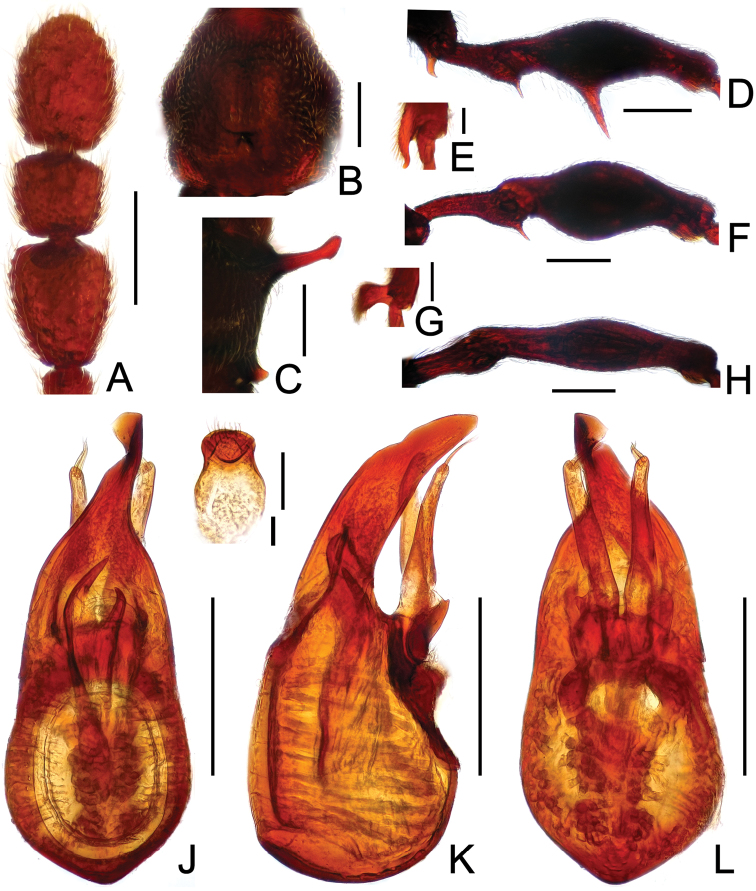
Diagnostic features of *Pselaphodes tibialis*. **A** antenna **B** pronotum **C** median meteventral process, in lateral view **D** procoxa, protrochanter and profemur **E** apical portion of protibia **F** mesotrochanter and mesofemur **G** apical portion of mesotibia **H** metatrochanter and metafemur **I** sternite IX **J** aedeagus, in dorsal view **K** same, in lateral view **L** same, in ventral view. Scales (mm): **A, B, C, D, F, H, J, K, L** = 0.2; **I** = 0.1; **E, G** = 0.05.

Male (Fig. 15B). Length 2.52–2.58. Head longer than wide, HL 0.58–0.59, HW 0.54–0.55; eyes each composed of about 40 facets. Antennal clubs as in Fig. 17A. Pronotum (Fig. 17B) about as long as wide, PL 0.54–0.55, PW 0.54–0.56, with lateral margins slightly angularly expanded laterally. Elytra wider than long, EL 0.68–0.71, EW 0.99–1.00. Long metaventral (Fig. 17C) processes broadened apically. Procoxae with sharp ventral tooth, protrochanters with short thin ventral spine, profemora with long sharp spine at ventral margin (Fig. 17D), protibiae with distinct apical spur (Fig. 17E); mesotrochanters with small spine at ventral margin, mesofemora simple (Fig. 17F), mesotibiae (Fig. 17G) with big apical projection; metatrochanters and metafemora (Fig. 17H) simple. Abdomen broad at base and narrowed apically, AL 0.72–0.73, AW 1.00–1.02. Sternite IX as in Fig. 17I. Aedeagus length 0.53, with asymmetric median lobe (Figs 17J–L).

**Female.** Unknown.

#### Comparative notes.

The resemblance in general habitus, antennal modification, placement of spines on the legs, and the shared modified pro- and mesotibiae place *Pselaphodes tibialis* closest to *Pselaphodes venustus* sp. n. described below. The two species can be separated by the smaller body size, the metaventral process being much thinner, and different aedeagal form in *Pselaphodes tibialis*, while *Pselaphodes venustus* is larger in size (3.07–3.34) and the metaventral process are much stouter.

#### Distribution.

Southwest China: Yunnan.

#### Biology.

Individuals were sifted from mixed leaf litter in a ravine.

#### Etymology.

The specific name refers to the modifications present on the pro- and mesotibiae.

### Pselaphodes venustus

Yin & Li sp. n.

urn:lsid:zoobank.org:act:892D0215-8B5D-4CE0-8CDA-6BE661BC1414

http://species-id.net/wiki/Pselaphodes_venustus

Figs 15C 18

#### Type materia

(1 ♂, 1 ♀)**.** Holotype: ♂, labeled ‘CHINA (Yunnan) Dali Bai Aut. Pref., Jizu Shan, summit plateau, / 37 km NE Dali 3150 m, (mixed / forest, sifted from litter, moss) / 25°58'30"N, 100°21'36"E / 5.IX.2009 DW Wrase [28]’ (cSch). Paratype: 1 ♀, same label data, except ‘leg. M. Schülke [CH09-28]’ (cSch).

#### Diagnosis.

Reddish brown; length 3.07–3.34; postgenae rounded; antennomeres IX–XI enlarged, IX modified in the male; pronotum with lateral margins roundly expanded laterally; with stout metaventral processes apically broadened; metacoxae simple; aedeagus with asymmetric median lobe.

#### Description.

**Figure 18.**
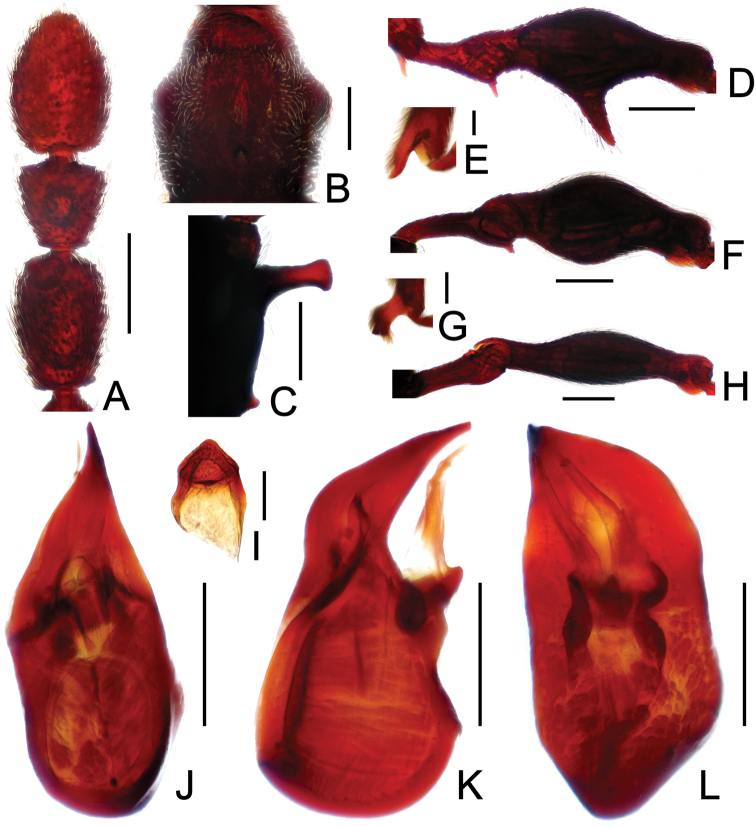
Diagnostic features of *Pselaphodes venustus*. **A** antenna **B** pronotum **C** median meteventral process, in lateral view **D** procoxa, protrochanter and profemur **E** apical portion of protibia **F** mesotrochanter and mesofemur **G** apical portion of mesotibia **H** metatrochanter and metafemur **I** sternite IX **J** aedeagus, in dorsal view **K** same, in lateral view **L** same, in ventral view. Scales (mm): **A, B, C, D, F, H, J, K, L** = 0.2; **I, G** = 0.1; **E** = 0.05.

Male (Fig. 17C). Length 3.07. Head slightly longer than wide, HL 0.65, HW 0.60; eyes each composed of about 30 facets. Antennal clubs as in Fig. 18A. Pronotum (Fig. 18B) slightly longer than wide, PL 0.65, PW 0.61, with lateral margins roundly expanded laterally. Elytra wider than long, EL 0.83, EW 1.16. Metaventral processes stout with enlarged apices (Fig. 18C). Procoxae with sharp ventral tooth, protrochanters with short thin ventral spine, profemora with large spine at ventral margin (Fig. 18D), protibiae with distinct apical spur (Fig. 18E); mesotrochanters with small spine at ventral margin, mesofemora simple (Fig. 18F), mesotibiae (Fig. 18G) with big apical projection; metatrochanters and metafemora (Fig. 18H) simple. Abdomen broad at base and narrowed apically, AL 0.94, AW 1.20. Sternite IX as in Fig. 18I. Aedeagus length 0.65, with asymmetric median lobe (Figs 18J–L).

Female. Similar to male in general; BL 3.34, HL 0.68, HW 0.63, PL 0.65, PW 0.60, EL 0.83, EW 1.19, AL 1.18, AW 1.34. Eyes each composed of about 30 facets. Metaventral processes absent.

#### Comparative notes.

The differences in body size and forms of the tibial modifications between *Pselaphodes venustus* and *Pselaphodes tibialis* were thought to be intraspecific variation before suspicions arose, and dissections of the genital segments of both species were done. Now it is clear that *Pselaphodes venustus* represents a distinct species. It can be readily separated from *Pselaphodes tibialis* by the larger size, the much stouter metaventral processes and, primarily, the aedeagal form.

#### Distribution.

Southwest China: Yunnan.

#### Biology.

Species were sifted from leaf litter and moss in a mixed forest.

#### Etymology.

The Latin word ‘*venustus*’ means ‘attractive in appearance’, with regard to the strong modifications of the pro- and mesotibiae.

### Pselaphodes zhongdianus

Yin & Li sp. n.

urn:lsid:zoobank.org:act:875C80DE-D055-4A46-8769-F502AA91A5D4

http://species-id.net/wiki/Pselaphodes_zhongdianus

Figs 15D 19

#### Type material

(1 ♂)**.** Holotype: ♂, labeled ‘CHINA: N-Yunnan [C03-05] / Zhongdian Co., 46 km SSE / Zhongdian, 27°27.0'N, 99°54.7'E, / 3050-3100 m, creek valley, secondary / mixed forest, bamboo, mushrooms, / 17.VIII.2003, leg. M. Schülke’ (cSch).

#### Diagnosis.

Reddish brown; length 3.23; postgenae round laterally; antennomeres IX–XI enlarged, IX modified in the male; pronotum with lateral margins slightly angularly expanded laterally; with metaventral processes apically narrowed; metacoxae simple; aedeagus with asymmetric median lobe.

#### Description.

Male (Fig. 15D). Length 3.23. Head longer than wide, HL 00.73, HW 0.70; eyes each composed of about 45 facets. Antennal clubs as in Fig. 19A. Pronotum (Fig. 19B) about as long as wide, PL 0.72, PW 0.71, with lateral margins slightly angularly expanded laterally. Elytra wider than long, EL 0.89, EW 1.37. Metaventral (Fig. 19C) processes narrowed apically. Protrochanters with short ventral spine, profemora with long sharp spine at ventral margin (Fig. 19D), protibiae with tiny apical spur (Fig. 19E); mesotrochanters with two small spines at ventral margin, mesofemora simple (Fig. 19F); metatrochanters and metafemora (Fig. 19G) simple. Abdomen broad at base and narrowed apically, AL 0.89, AW 1.44. Sternite IX as in Fig. 19H. Aedeagus length 0.65, with asymmetric median lobe (Figs 19I–K).

**Female.** Unknown.

#### Comparative notes.

Placed near *Pselaphodes erlangshanus* and *Pselaphodes flexus* as discussed above, readily separated from both species by the unique antennomeres IX being strongly roundly projecting at the anterolateral margin.

#### Distribution.

Southwest China: Yunnan.

#### Biology.

Individuals was sifted from bamboo leaf litter and mushrooms in a secondary mixed forest**.**

#### Etymology.

The new species is named after the type locality, Zhongdian.

##### Unassociated female specimens

**Notes.** Only the collecting data are cited here, and all specimens bear an identification label as the following, so that they can be tracked by future workers: ‘Unassociated ♀ sp. xx [number] / *xxx* [genus name] sp. / det. Yin & Li, 2012’. Fourteen of the following species are represented by single specimens (all in cSch).

**Figure:**
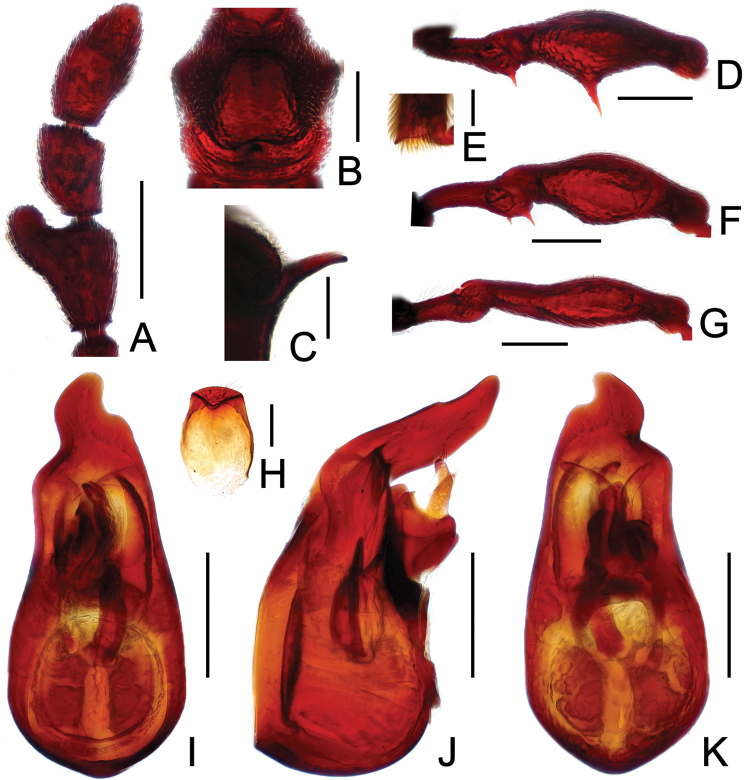
**Figure 19.** Diagnostic features of *Pselaphodes zhongdianus*. **A** antenna **B** pronotum **C** median meteventral process, in lateral view **D** protrochanter and profemur **E** apical portion of protibia **F** mesotrochanter and mesofemur **G** metatrochanter and metafemur **H** sternite IX **I** aedeagus, in dorsal view **J** same, in lateral view **K** same, in ventral view. Scales (mm): **A, B, D, F, G** = 0.3; **C, I, J, K** = 0.2; **H, E** = 0.1.

##### *Labomimus*:

sp. 1. 1 ♀, labeled ‘CHINA: W-Sichuan (6) / Daxue Shan, Paoma-Shan / b. kangding, 30.02.56N, / 101.58.05E, 2700-2900 m / 22.05.1997, M. Schülke’.

sp. 2. 1 ♀, labeled ‘CHINA:Yunnan, Nujiang Lisu Pref., / Gaoligong Shan, “Cloud pass” 3150 m / 21 km NW Liuku (shrubs, / Vaccinium, bamboo, litter sifted) / 25°58'21"N, / 98°41'01"E / 2.IX.2009 D.W. Wrase [22A]’.

sp. 3. 1 ♀, labeled ‘CHINA (Yunnan) / Baoshan Pref., mount. range / 22 km S Tengchong, 1750 m / 24°49'29"N, 98°29'27"E / (loamy banks of fishponds) / 2.VI.2007 D.W. Wrase [18]’.

sp. 4. 1 ♀, labeled ‘CHINA W. Sichuan (Aba / Tibet. Aut. Pref., Weizhou Co.) / Quilonglai Shan, Wolong valley / 69 km WSW Dujiangyan, 3500 m / 30°54'N, 102°59'E (mix. forest) / 15.VIII.1999 D.W. Wrase’.

sp. 5. 1 ♀, labeled ‘CHINA: W-Sichuan 1999 / Ya’an Prefcture Fulin Co. / Daxiang Ling, Rd., zw. Hanyuanjie u. / Siping. 51 km NNE Shimian, 2000 m / 29°39N, 102°37E, Ufer, Gesiebe / 10. VII., leg. M. Schülke’.

sp. 6. 1 ♀, labeled ‘CHINA: S-Shaanxi (Daba Shan) / NW pass 25 km NW Zhenping, / 32°01N, 109°19E, / 2150 m, 11.VII.2001, / leg. M. Schülke [01-09] // creek valley, young coniferous / forest, moss (sifted) [01-09]’.

sp. 7. 1 ♀, labeled ‘CHINA: Yunnan, Dali Bai Aut. Pref., / Zhemo Shan, 7 km SW Xiaguan, / 25°32–33'N, 100°10-11'E, 2870–2970 m, / scrub with bamboo, oaks & / Rhododendr., litter sifted, 18. IX. / 2009, leg. M. Schülke [09-60]’.

sp. 8. 1 ♀, labeled ‘CHINA: N-Yunnan [C03-15] / Dali Bai Nat. Aut. Pref., Diancang Shan, / 5 km SSW Dali old town, creek valley / above cablecar, 25°38.7'N, 100°08.3'E, / shrub, bamboo, moss, old flood debris, / 2800 m, 26.VIII.2003, M. Schülke’.

sp. 9. 1 ♀, labeled ‘CHINA: W-Sichuan 1999 / Ganzi Tibet. Aut. Pref., Luding Co. / W Erlangshan-pass, 2600 m / 7 km SSE Luding, 25°51'N, / 102°15'E, Laubstreu, Pilze / 29.VI., leg. M. Schülke’; 1 ♀, labeled ‘CHINA: W-Sichuan 1999 / Ya’an Prefecture, Tianquan Co. / E Erlang Shan Pass, 2900 m, / 9 km SE Luding, 29°52'N, / 102°18'E, Gesiebe / 20.VI., leg. M. Schülke’.

##### *Pselaphodes*:

sp. 10 1 ♀, labeled ‘CHINA: Yunnan, Pu’er Pref., / Ailao Shan, 37 km NW Jingdong, / 24°45'12"N, 100°41'24.5"E, 2300 m, / devastated forest remnant, litter & / dead wood sifted, 13.IX.2009, / leg. M. Schülke [CH09-48]’.

sp. 11. 1 ♀, labeled ‘CHINA: Yunnan, Lincang Pref., / Xue Shan, 48 km N Lincang, / 2070 m, 24°16'03"N, 100°07'13"E, / forest remnant, N-slpoe, litter & / mushrooms sifted, 12.IX.2009, leg. M. Schülke [CH09-48]’.

sp. 12. 1 ♀, labeled ‘CHINA (N-Yunnan) Lijiang Naxi / Aut. Co., 3 km NW Yongsheng / 53 km WSW Lijiang, 1950-2000 m / 29°41'08"N, 100°43'1"E (SE-slope, / secondary broadleaved forest) / 14.VIII.2003 Wrase [03]’.

sp. 13. 1 ♀, labeled ‘CHINA: W-Sichuan 1999 / Ya’an Prefecture, Tianquan Co. / Jiajin Shan, Tal oberh. Labahe / N.R.St., 57 km W Ya’an, 30°06'N, 102°25'E, Streu, Rinde, Pilze, 1800 m / 12.VII., leg. M. Schülke’.

sp. 14. 1 ♀, labeled ‘CHINA: Zhejiang [CH07-37], Tianmu / Shan, pass 25 km NNW Linanm 620-820 / m, 30°25'10"N, 119°35'30"E, creek / valley with bamboo and mixed forest, / litter, sifted, 16.VI.2007, M. Schülke’.

sp. 15. 1 ♀, labeled ‘CHINA: N-Yunnan [C03-05] / Zhongdian Co., 46 km SSE / Zhongdian, 27°27.0'N, 99°54.7'E, / 3050-3100 m, creek valley, secondary / mixed forest, bamboo, mushrooms, / 17.VIII.2003, leg. M. Schülke’.

sp. 16. 2 ♀♀, labeled ‘China: Shaanxi, Qin Ling Shan / 110.06E, 34.27N / Hua Shan Mt. N Valley, 1200- / 1400 m, 118 km E Xi’an, sifted / 18.-20.08.1995, leg. M. Schülke’.

## Supplementary Material

XML Treatment for Labomimus cognatus

XML Treatment for Labomimus dabashanus

XML Treatment for Labomimus mirus

XML Treatment for Labomimus paratorus

XML Treatment for Labomimus sarculus

XML Treatment for Labomimus schuelkei

XML Treatment for Labomimus vespertilio

XML Treatment for Linan tendothorax

XML Treatment for Pselaphodes distincticornis

XML Treatment for Pselaphodes erlangshanus

XML Treatment for Pselaphodes flexus

XML Treatment for Pselaphodes jizushanus

XML Treatment for Pselaphodes nomurai

XML Treatment for Pselaphodes tibialis

XML Treatment for Pselaphodes venustus

XML Treatment for Pselaphodes zhongdianus
